# Schwann Cell‐Specific Ablation of Beclin 1 Impairs Myelination and Leads to Motor and Sensory Neuropathy in Mice

**DOI:** 10.1002/advs.202308965

**Published:** 2024-12-16

**Authors:** Lisa Gambarotto, Loris Russo, Silvia Bresolin, Luca Persano, Rachele D'Amore, Giulia Ronchi, Federica Zen, Luisa Muratori, Alice Cani, Samuele Negro, Aram Megighian, Sonia Calabrò, Paola Braghetta, Dario Bizzotto, Matilde Cescon

**Affiliations:** ^1^ Department of Molecular Medicine University of Padova Via U. Bassi 58/B Padova 35131 Italy; ^2^ Department of Biology University of Padova Via U. Bassi 58/B Padova 35131 Italy; ^3^ Department of Women and Children's Health University of Padova via Giustiniani 3 Padova 35127 Italy; ^4^ Istituto di Ricerca Pediatrica – Città della Speranza Corso Stati Uniti 4 Padova 35128 Italy; ^5^ Department of Clinical and Biological Sciences & Neuroscience Institute Cavalieri Ottolenghi (NICO) University of Torino Regione Gonzole 10, Orbassano Torino 10043 Italy; ^6^ U.O.C. Clinica Neurologica Azienda Ospedale‐Università Padova Via Giustiniani 5 Padova 35128 Italy; ^7^ Department of Biomedical Sciences University of Padova Via U. Bassi 58/B Padova 35131 Italy; ^8^ Padova Neuroscience Center University of Padova Via G. Orus, 2 Padova 35131 Italy

**Keywords:** autophagy, Beclin 1, motor function, myelination, neuropathy, Schwann cells, sensory function

## Abstract

The core component of the class III phosphatidylinositol 3‐kinase complex, Beclin 1, takes part in different protein networks, thus switching its role from inducing autophagy to regulating autophagosomal maturation and endosomal trafficking. While assessed in neurons, astrocytes, and microglia, its role is far less investigated in myelinating glia, including Schwann cells (SCs), responsible for peripheral nerve myelination. Remarkably, the dysregulation in endosomal trafficking is emerging as a pathophysiological mechanism underlying peripheral neuropathies, such as demyelinating Charcot‐Marie‐Tooth (CMT) diseases. By knocking out Beclin 1 in SCs here a novel mouse model (*Becn1* cKO) is generated, developing a severe and progressive neuropathy, accompanied by involuntary tremors, body weight loss, and premature death. Ultrastructural analysis revealed abated myelination and SCs displaying enlarged cytoplasm with progressive accumulation of intracellular vesicles. Transcriptomic and histological analysis from sciatic nerves of 10‐day and 2‐month‐old mice revealed pro‐mitotic gene deregulation and increased SCs proliferation at both stages with axonal loss and increased immune infiltration in adults, well reflecting the progressive motor and sensory functional impairment that characterizes *Becn1* cKO mice, compared to controls. The study establishes a further step in understanding key mechanisms in SC development and points to Beclin 1 and its regulated pathways as targets for demyelinating CMT forms.

## Graphical abstract

Beclin 1, as a component of the class III‐phosphatidylinositol 3‐kinase complex, is involved in autophagy induction as well as in autophagosome maturation and endosomal trafficking. This study demonstrates the essential role of Beclin 1‐regulated pathways in peripheral myelination. In vivo Schwann cell‐specific Beclin 1 ablation leads to defective peripheral myelination and abnormal radial sorting in turn resulting in a severe and progressive early‐onset sensorimotor neuropathy in mice.

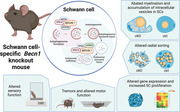

## 1 Introduction

Schwann cells (SCs) are the most abundant population of glial cells in the peripheral nervous system (PNS), responsible for myelin production. Far from providing a mere supportive role to neurons, SCs are fundamental for proper PNS development and function.^[^ ^1^, ^2^ ^]^ SC precursors, derived from neural crest cells, differentiate into immature SCs in late embryonic stages, receiving signals released by developing axons, like Neuregulin 1 type III (NRG1‐III),^[^ ^3^, ^4^ ^]^ and then, in the early postnatal phase, they coordinate a process called radial sorting.^[^ ^5^ ^]^ Immature SCs, associating to and segregating axons larger than 1 µm in diameter at the periphery of bundles of growing axons, will acquire a 1:1 relationship with such large caliber axons, and will differentiate into pro‐myelinating SCs.^[^ ^5^ ^]^ On the other hand, immature SC will differentiate toward a non‐myelinating cell fate surrounding bundles of small caliber axons, including the C‐fiber nociceptors, the postganglionic sympathetic fibers, and some of the preganglionic sympathetic and parasympathetic fibers.^[^ ^6^ ^]^ These processes are tightly regulated by integrated signals. In particular, promyelinating SCs receive signals by both the axon and own basal lamina, resulting in the promotion of cell‐cycle exit and the cytoskeletal reorganization necessary for radial sorting and myelination initiation.^[^ ^7^ ^]^ Among others, the formation of phosphatidylinositol 3‐phosphate (PI3P), guided by class III‐phosphatidylinositol 3‐kinase (PIK3C3) activation, contributes to polarizing SCs and regulating membrane protrusion and wrapping.^[^ ^8^ ^]^ During their differentiation and function SCs undergo robust morphological and functional changes, which include membrane compaction and abundant lipid and protein production. It was shown that autophagy in these cells is fundamental for proper PNS development, promoting myelin compaction through the removal of SC cytoplasm during development,^[^ ^9^ ^]^ for membrane specification/recycling and for the maintenance of peripheral nerve homeostasis.^[^ ^10^ ^]^ Moreover, in the last years, a disruption of the autophagic flux as well as a dysregulation in endosomal sorting and related signaling have emerged as potential pathophysiological mechanisms underlying the most common form of inherited peripheral neuropathies, known as Charcot‐Marie‐Tooth (CMT) disease.^[^ ^11^ ^]^ Genes directly or indirectly impacting such processes, like the ones involved in phosphoinositide maintenance and homeostasis, were found mutated in either axonal (affecting the axons of peripheral neurons) or demyelinating (primarily affecting SCs) forms of CMT, although a thorough understanding of the molecular pathways involved is still missing.^[^ ^12^, ^13^, ^14^ ^]^

Particularly important in these pathways and never investigated so far in the context of SC differentiation and homeostasis is Beclin 1 protein. Beclin 1, the orthologue of yeast Atg6, is a core component of the PIK3C3 complex and, thanks to its involvement in the controlled production of PI3P via PI3KC3 activation, Beclin 1 exerts a crucial function in the regulation of membrane dynamics. Indeed, a role for Beclin 1 was described in vesicle formation, trafficking, and fusion, participating in the regulation of different autophagy steps and also in endocytosis, phagocytosis, vesicles transport, and membrane delivery.^[^ ^15^, ^16^, ^17^, ^18^ ^]^ Beclin 1 dysregulation is involved in several disorders, most notably cancer,^[^ ^19^, ^20^ ^]^ heart disease,^[^ ^21^ ^]^ and neurodegenerative diseases such as Alzheimer's, Huntington's and Parkinson's disease, and amyotrophic lateral sclerosis.^[^ ^22^, ^23^ ^]^ Nonetheless, since autophagy has increasingly become a key pathway in inherited peripheral neuropathies,^[^ ^11^ ^]^ the pivotal role of Beclin 1 in regulating autophagy and the endocytic pathway is expected to have a relevant impact in this context.

Since whole‐body *Becn1* homozygous null mice die early in embryogenesis,^[^ ^24^ ^]^ here we generated a conditional gene knockout specifically in SCs, to investigate the role that Beclin 1 exerts in these cells and in myelination. The newly generated mouse line resulted in a model for severe and progressive peripheral neuropathy, displaying involuntary tremors, progressive hindlimb paresis, decreased body weight, and diminished lifespan. Together with defective myelination, we described radial sorting defects, motor and sensory neuron impairments, immune infiltrate in adults, and a huge transcripts deregulation. Among the altered pathways at the basis of the observed severe neuropathic phenotype, a strong deregulation of the PI3K/AKT/mTOR pathway highlighted how Beclin 1 is able to coordinate integrated signaling pathways essential for SC maturation and nerve development and homeostasis.

## 2 Results

### 2.1 Generation and Validation of a Novel Schwann Cell‐Specific *Becn1* Knockout Mouse Model

**Figure 1:**
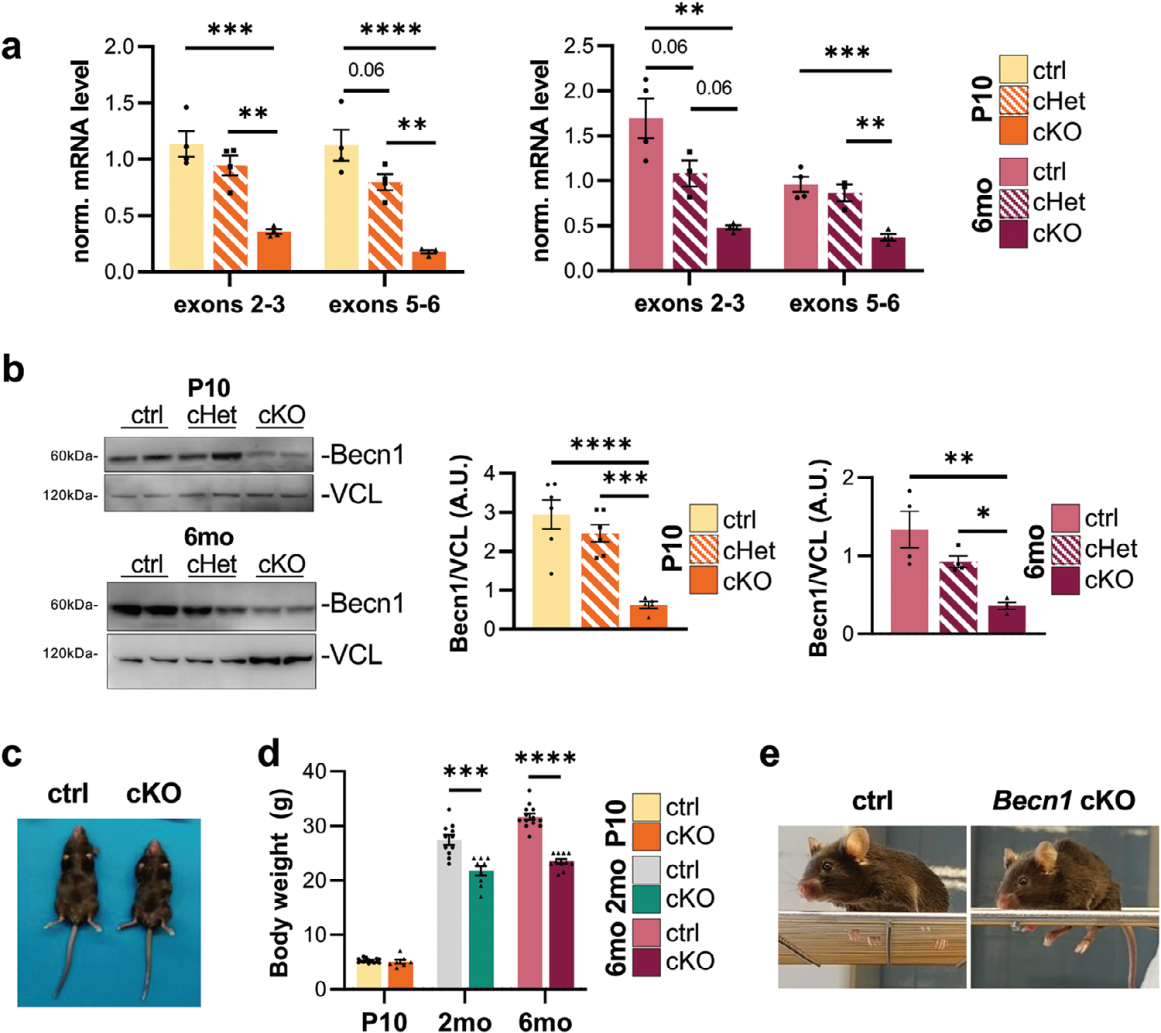
a) Normalized mRNA levels from RT‐qPCR analyses on two different regions (exons 2–3 and exons 5–6) of *Becn1* transcript in sciatic nerve of P10 and 6‐month‐old control, *Becn1* cHet and *Becn1* cKO mice (*n* = 3–4 mice each group; ^**^ *P* < 0.01; ^***^ *P* < 0.001; ^****^ *P*<0.0001; Ordinary one‐way ANOVA with Tukey's multiple comparison test). b) Representative western blot images and relative densitometric quantifications of Beclin 1 (Becn1) on total sciatic nerves protein extracts of P10 and 6‐month‐old control, *Becn1* cHet and *Becn1* cKO mice. Vinculin (VCL) was used as a loading control (*n* = 4–6 mice each group; ^*^ *P* < 0.05; ^**^ *P* < 0.01; ^***^ *P* < 0.001; ^****^ *P* < 0.0001; Ordinary one‐way ANOVA with Tukey's multiple comparison test). c) Representative ventral view of 2‐month‐old control and *Becn1* cKO mice. d) Body weight (g) of male control and *Becn1* cKO mice at P10 and at 2 and 6 months of age (*n* = 7–15 mice each group; ^***^ *P* < 0.001 ^****^ *P* <0.0001; Student's *t* test). e) Pictures of 6‐month‐old control and *Becn1* cKO mice, representing the inability to normally grab and stand on a grid of the adult *Becn1* cKO mice. 2 mo, 2‐month‐old; 6 mo, 6‐month‐old; ctrl, control; A.U., arbitrary units.

To inactivate *Becn1* gene only in SCs, we crossed *Becn1^fl/fl^* mice, bearing *LoxP* sites flanking exons 4 to 7 of *Becn1* gene, with mice expressing Cre recombinase under the control of *Myelin protein zero* (*Mpz*) promoter, active in SCs since embryonic stage E13.5‐E14.5 (see Experimental Section and Figure S1a, Supporting Information).^[^ ^25^ ^]^ First, we validated the expected deletion of *Becn1* exons in SCs from the conditional knockout mice (*Becn1* cKO), by analyzing Beclin 1 transcript and protein levels in sciatic nerve extracts. *Becn1* transcript was significantly reduced in 10‐day‐ and 6‐month‐old *Becn1* cKO mice when compared with control and heterozygous (*Becn1* cHet) littermates. This was evident when considering exons in the site of deletion (exons 5–6) as well as exons upstream to the deletion site (exons 2–3), suggesting the occurrence of nonsense‐mediated decay on *Becn1* cKO transcripts (**Figure** **1**a). Moreover, we confirmed the significant reduction of Beclin 1 protein level in extracts from whole sciatic nerve from 10‐day‐ and 6‐month‐old *Becn1* cKO mice, when compared with control and *Becn1* cHet littermates (Figure 1b). As expected, a residual amount of Beclin 1 protein was detected, due to the contribution of other cell types, including axons, fibroblasts, and endothelial cells, in the protein lysates derived from sciatic nerves. To further confirm that Beclin 1 ablation occurred specifically in the myelinating glia of the PNS and not in the central nervous system, we verified that Beclin 1 transcript and protein levels were not altered in mRNA and protein extracts from the cerebellum (Figure S1b,c, Supporting Information). Altogether these data confirmed the correct ablation of Beclin 1 in the myelinating glia of the PNS.

### 2.2 *Becn1* cKO Displayed a Severe Neuropathic Phenotype

The effects of Beclin 1 ablation in SCs were remarkably severe and overtly evident even at the first gross phenotypic evaluation. Since weaning, *Becn1* cKO mice displayed distinctive involuntary tremors already in resting conditions, with progressive motor impairment, particularly affecting posterior legs, leading to a prominent hindlimb paresis by the age of 6 months (representative videos of 2‐ and 6‐month‐old animals in Videos S1–S3, Supporting Information). This neuropathic condition was also accompanied by an overall smaller size (Figure 1c) and a marked body weight loss in adult *Becn1* cKO mice, when compared to control littermates (Figure 1d). Moreover, *Becn1* cKO mice progressively lost the ability to walk properly, with a noticeable inability to grab or stand on a grid (Figure 1e). 7 months of age was defined as the humane endpoint for *Becn1* cKO mice. Taken together these observations revealed that the lack of Beclin 1 in SCs led to a progressive and severe peripheral neuropathic phenotype, characterized by early onset of symptoms and a rapid progression, resulting in premature death.

### 2.3 Lack of Beclin 1 in SCs Impairs Myelination

**Figure 2:**
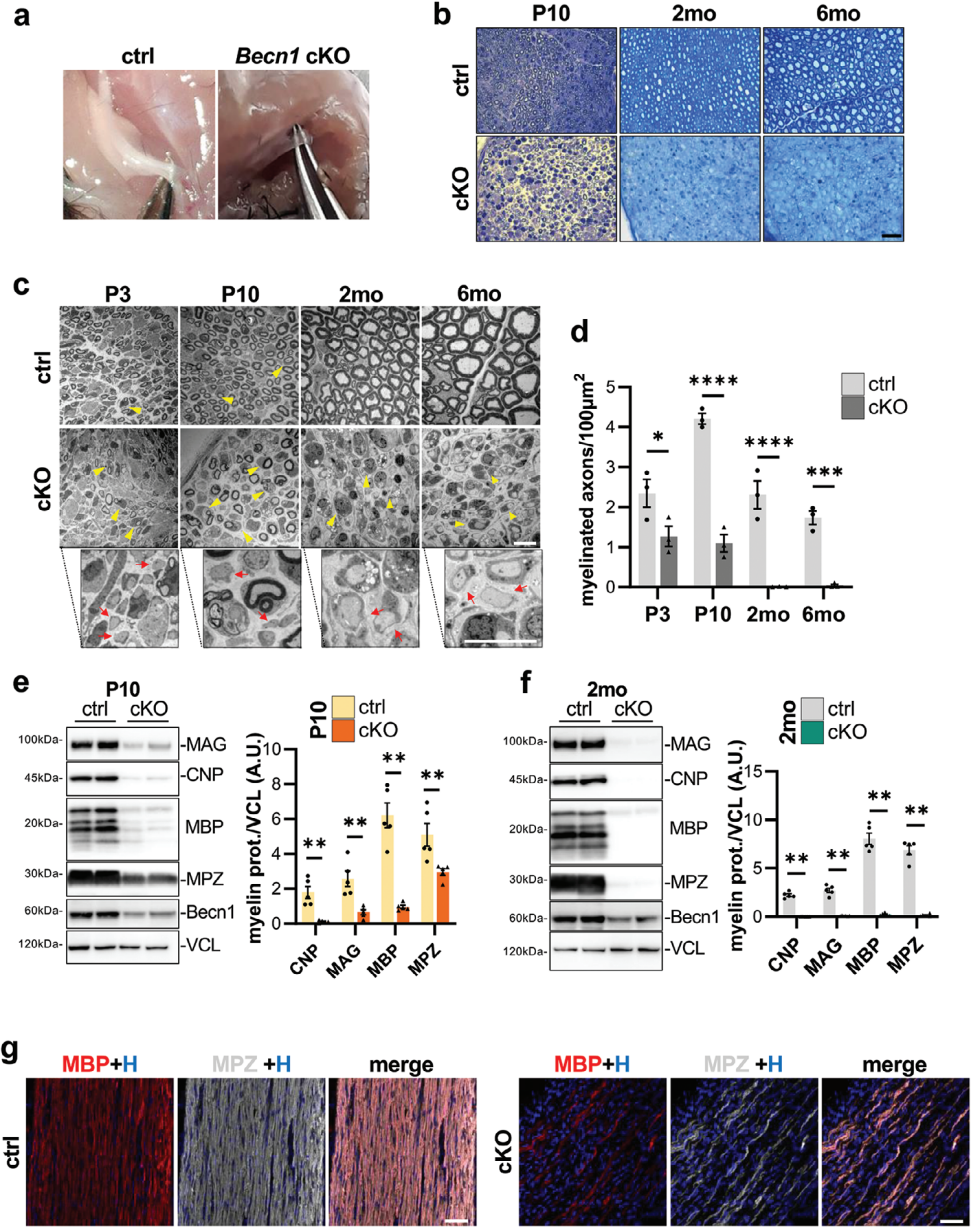
a) Representative images of exposed sciatic nerve during dissection of 2‐month‐old mice, showing the translucent and transparent appearance of *Becn1* cKO nerves when compared to the white appearance of nerves of control mice. b) Representative images of toluidine blue‐stained semithin sciatic nerve transversal sections from P10, 2‐month‐old, and 6‐month‐old control and *Becn1* cKO mice. Scale bar = 40 µm. c) Representative transmission electron microscopy images of sciatic nerve transversal sections from P3, P10, 2‐ and 6‐month‐old control and *Becn1* cKO mice. Arrowheads indicate enlarged SC cytoplasm. Enlargements are provided for cKO representative images, showing the presence of naked axons (red arrows). Scale bar = 10 µm. d) Quantification of the number of myelinated axons per 100 µm^2^ of area manually counted from images as in (c) (*n* = 3 mice each group; ^*^ *P* < 0.05; ^***^ *P* < 0.001; ^****^ *P* < 0.0001; 2‐way ANOVA with Holm‐Šídák's multiple comparisons test). e,f) Representative western blot images and relative densitometric quantifications of myelin proteins on total median nerves protein extracts of P10 (e) and 2‐month‐old (f) control and *Becn1* cKO mice. Vinculin (VCL) was used as a loading control (*n* = 5–6 mice each group; ^**^ *P* < 0.01; Mann‐Whitney U test). g) Representative confocal immunofluorescence images for myelin basic protein (MBP, red) and myelin protein zero (MPZ, grey) on longitudinal cryosection of sciatic nerves from P10 control and *Becn1* cKO mice. Nuclei were counterstained with Hoechst (H, blue). Scale bar = 50 µm. 2 mo, 2‐month‐old; 6 mo, 6‐month‐old; ctrl, control; A.U., arbitrary units.

Upon nerve dissection, at a gross examination, *Becn1* cKO sciatic nerves appeared thinner and more translucent when compared to control littermates (**Figure** **2**a), pointing to a major myelination defect. To better characterize such defect, we analyzed toluidine blue‐stained semi‐thin sciatic nerve transversal sections of postnatal day 10 (P10) pups and 2‐ and 6‐month‐old animals. Although nerves from control pups displayed signs of ongoing myelination, including abundant SC cytoplasm and not fully compacted and completed myelination, Beclin 1‐deficient nerves appeared already noticeably altered, displaying SCs with highly enlarged cytoplasm and only few myelinated axons (Figure 2b). In addition, 2‐ and 6‐month‐old *Becn1* cKO samples displayed much more severe phenotypic defects, with an almost complete absence of myelinated axons when compared to age‐matched controls (Figure 2b). Of note, the sciatic nerves of *Becn1* cHet animals appeared normal, being undistinguishable from age‐matched control samples (Figure S2a, Supporting Information). Ultrastructural analysis of sciatic nerves by transmission electron microscopy (TEM) further confirmed the early onset and progressive worsening of the phenotypic defects observed in *Becn1* cKO mice, unraveling that such impairments are already detectable also in P3 *Becn1* cKO pups (Figure 2c; Figure S2b, Supporting Information). Nonetheless, small and large caliber naked axons are evident throughout all the analyzed timepoints in cKO sciatic nerves (Figure 2c). The quantification of the number of myelinated axons per area highlighted this evidence, showing significantly reduced myelination in P3 *Becn1* cKO pups, ≈75% less myelinated axons per area in P10 *Becn1* cKO pups, and almost complete absence of myelination in 2‐ and 6‐month‐old *Becn1* cKO mice with respect to controls (Figure 2d). Interestingly, western blot experiments on median nerve protein extracts revealed that various myelin‐associated protein amounts, including myelin basic protein (MBP), myelin‐associated glycoprotein (MAG), myelin protein zero (MPZ) and 2′,3′‐Cyclic‐nucleotide 3′‐phosphodiesterase (CNP), were very strongly decreased in P10 *Becn1* cKO pups compared to controls (Figure 2e), and almost completely absent in 2‐ and 6‐month‐old *Becn1* cKO animals compared to controls (Figure 2f) and *Becn1* cHet (Figure S2c, Supporting Information). In agreement with the above data, immunofluorescence analysis of MBP and MPZ protein distribution in longitudinal sciatic nerve sections of P10 mice confirmed a dramatic decrease in myelin‐related proteins in mutant mice (Figure 2g). A more significant reduction in myelin staining was highlighted by immunofluorescence experiments against MBP in entire transversal sciatic nerve cryosections of 2‐month‐old *Becn1* cKO mice compared to controls (Figure S2d, Supporting Information). Importantly, transversal TEM optic nerve images showed that *Becn1* cKO nerves of the central nervous system were unaltered (Figure S2e, Supporting Information), and also the expression of myelin proteins in the cerebellum was unaffected in *Becn1* cKO samples (Figure S1c, Supporting Information), confirming that the defects were restricted to peripheral nerves, according to Cre recombinase expression. Altogether, these findings indicated that Beclin 1 deletion in SCs impairs myelination soon after birth, progressively worsening toward an almost complete absence of myelinated axons during adulthood, accompanied by the lack of myelin‐related proteins.

### 2.4 Beclin 1 Ablation Induces the Accumulation of Vesicles and Autophagy‐Related Proteins in SCs

**Figure 3:**
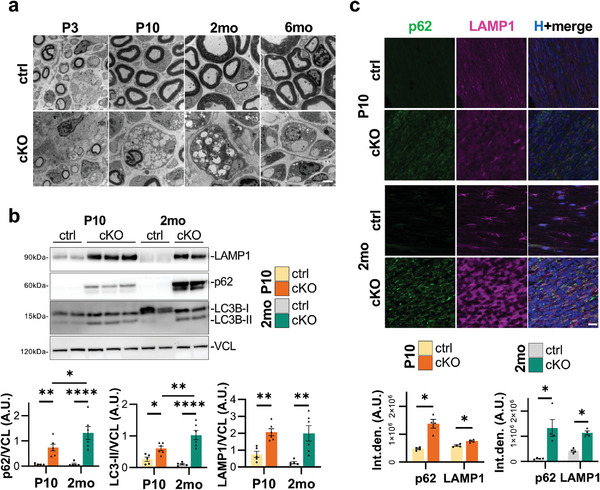
a) Representative high magnification transmission electron microscopy images of sciatic nerve transversal sections from P3, P10, 2‐month‐old and 6‐month‐old control and *Becn1* cKO mice. Scale bar = 2 µm. b) Representative western blot images and relative densitometric quantifications of the autophagy‐related proteins LAMP1, p62, and LC3B on total sciatic nerves protein extracts of P10 and 2‐month‐old control and *Becn1* cKO mice. Vinculin (VCL) was used as loading control (*n* = 5–6 mice each group; ^*^ *P* < 0.05; ^**^ *P* < 0.01; ^****^ *P* < 0.0001; 2‐way ANOVA with Holm‐Šídák's multiple comparisons test). c) Representative confocal immunofluorescence images and the relative integrated density quantification for p62 (green) and LAMP1 (magenta) on longitudinal cryosection of sciatic nerves from P10 and 2‐month‐old control and *Becn1* cKO mice. Nuclei were counterstained with Hoechst (H, blue); scale bar = 20 µm (*n* = 4 mice each group; ^*^ *P* < 0.05; Mann‐Whitney U test). 2 mo, 2‐month‐old; 6 mo, 6‐month‐old; ctrl, control; A.U., arbitrary units; Int.den., integrated density.

Beyond the remarkably affected myelination we observed, we further analyzed the ultrastructural appearance of *Becn1* cKO sciatic nerves. Starting already from P3 and P10 pups and worsening in adult mice, TEM images at higher magnification confirmed the presence of enlarged SC cytoplasm in *Becn1* cKO sciatic nerves, even when they are in a 1:1 association with axons. In addition, SCs displayed abnormal vesiculation, with a massive accumulation of intracellular material and organelles (**Figure** **3**a).

Considering the role of Beclin 1 in autophagy and vesicle trafficking regulation,^[^ ^26^ ^]^ we assessed the levels of some key autophagic and endosomal markers. First, western blot experiments revealed a remarkable accumulation of the autophagic receptor p62 in sciatic nerve protein extracts from P10 *Becn1* cKO pups compared to control mice, and even more evident in 2‐month‐old animals (Figure 3b), pointing to an increased requirement to degrade cytoplasmic material as well as to a diminished degradative competence of *Becn1* cKO SCs. p62 accumulation in *Becn1* cKO sciatic nerves was also confirmed by immunofluorescence analysis in longitudinal sections (Figure 3c), which appears consistent with p62‐positive dots marking at least some of the big vesicles observed in the ultrastructural micrographs. Beclin 1‐deficient sciatic nerves also showed a significant increase by western blot in the levels of the lipidated form of LC3B (LC3B‐II), marking autophagosomes, in P10 pups and significantly more in 2‐month‐old animals (Figure 3b). Moreover, concerning the late autophagic steps, the endo‐lysosomal marker LAMP1 was significantly upregulated in P10 and 2‐month‐old *Becn1* cKO sciatic nerves compared to control ones, as assessed both by western blot (Figure 3b) and immunofluorescence experiments (Figure 3c). Together these data underlined a clear autophagic impairment, compromising the ability to successfully process the degradative vesicles and contributing to their accumulation in the SC cytoplasm.

### 2.5 Transcriptional Analyses Revealed a Differential Impact of Beclin 1 Depletion at P10 and 2 Months of Age

**Figure 4:**
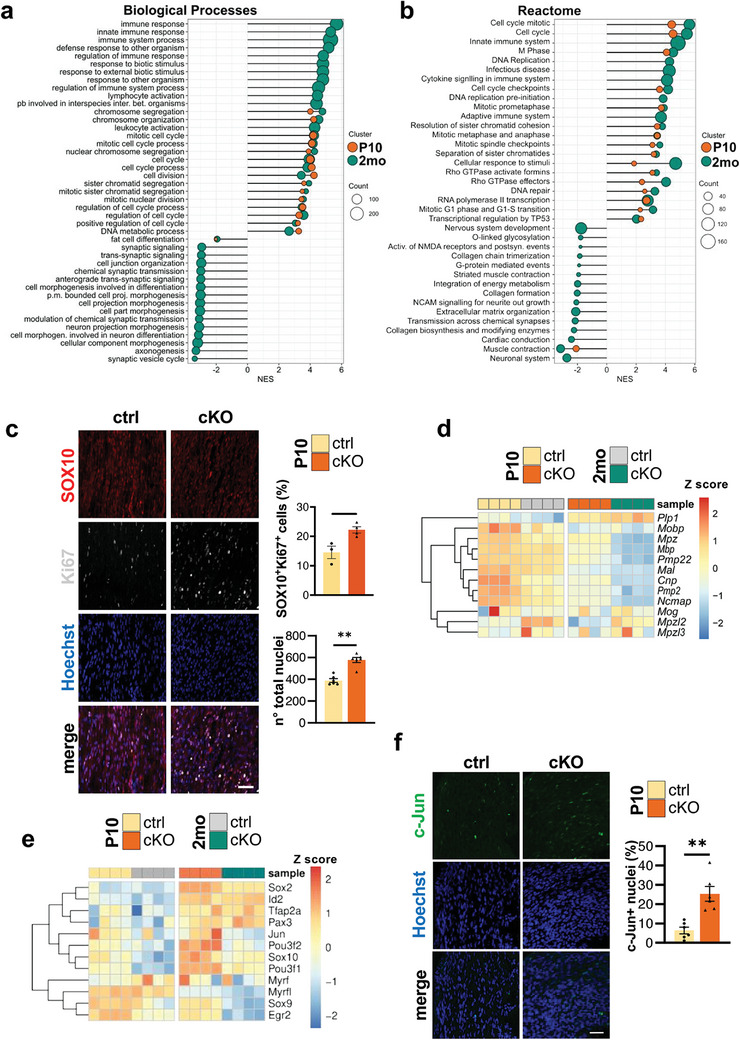
a,b) Dot plot summarizing the most significant positively (top 15) and negatively (top 15) enriched terms for Biological Processes Gene Ontology (a) and Reactome (b) in P10 and 2‐month‐old sciatic nerve DEGs. The size of the circle corresponds to the count of genes enriched in each gene sets. c) Representative confocal immunofluorescence images and the relative quantification of the percentage of both SOX10 (red) and Ki67 (grey) positive nuclei on total nuclei number, and of total nuclei number on longitudinal cryosection of sciatic nerves from P10 control and *Becn1* cKO mice. Nuclei were counterstained with Hoechst (blue); scale bar = 30 µm (*n* = 6 nerves sampled from 3 mice each group; ^*^ *P* < 0.05; ^**^ *P* < 0.01; Mann‐Whitney U test). d) Heatmap displaying the individual expression of myelin‐related genes. e) Heatmap displaying the individual expression of transcription factors involved in SC differentiation. f) Representative confocal immunofluorescence images and the relative quantification of the percentage of c‐Jun (green) positive nuclei on total nuclei number, on longitudinal cryosection of sciatic nerves from P10 control and *Becn1* cKO mice. Nuclei were counterstained with Hoechst (blue); scale bar = 50 µm (*n* = 6 nerves sampled from 4 mice each group; ^**^ *P* < 0.01; Mann‐Whitney U test). NES, Normalized Enrichment Score; 2 mo, 2‐month‐old; ctrl, control; A.U., arbitrary units.

To shed light on the processes involved in the development of the neuropathic phenotype observed in the *Becn1* cKO mouse line, we performed a transcriptomic analysis on sciatic nerve mRNA extracts from both P10 and 2‐month‐old control and *Becn1* cKO mice (Figure S3a, Supporting Information). The analysis described a profound transcriptional dysregulation, with 718 differentially expressed genes (DEGs) in P10 samples and 1851 DEGs in 2‐month‐old samples (Figure S3b,c, Supporting Information). In particular, the unique downregulated genes in *Becn1* cKO compared to controls were 146 in P10 mice and 476 in 2‐month‐old mice; 330 DEGs were shared between animals of both ages (Figure S3d, Supporting Information). Upregulated genes in *Becn1* cKO compared to controls were 32 in P10 mice, 825 in 2‐month‐old samples, and 210 shared by animals of both ages (Figure S3e, Supporting Information). The gene set enrichment analysis (GSEA) with both Gene Ontology for Biological Processes (**Figure** **4**a) and Reactome (Figure 4b), showed a strong positive enrichment at both ages of many genes related to cell cycle regulation and cell division in *Becn1* cKO samples compared with controls. Thus, we monitored cell proliferation by immunofluorescence on longitudinal sciatic nerve sections of P10 pups, finding a significant increase in the percentage of proliferating SCs (SOX10 and Ki67‐positive) in *Becn1* cKO mice compared to controls (Figure 4c). Not only the percentage of proliferating SCs were increased but also the number of total nuclei resulted significantly higher in *Becn1* cKO nerves compared to controls (Figure 4c), as well as it was qualitatively detectable also in samples of other ages (e.g., Figure 3c). These data support an increase in SCs proliferation when Beclin 1 is missing, from early time points onwards, well reflected by a marked decrease in the expression of late differentiation markers (Figure 4d). Interestingly, transcriptomic data also highlighted a clear deregulation of several transcription factors (TFs) directly modulating SC differentiation in *Becn1* cKO nerves compared to controls, maintaining SCs in an immature state (Figure 4e). SC precursors’ TFs including *Tfap2a* and *Pax3* ^[^ ^27^, ^28^ ^]^ resulted overexpressed in P10 and adult *Becn1* cKO nerves, whereas they were switched off in control ones, as expected. As well, *Pou3f1* and *Pou3f2*, expected to be downregulated in postnatal stages,^[^ ^29^ ^]^ were still upregulated in cKO nerves at P10. On the other hand, *Sox2* and *Id2*, TFs expressed by premyelinating SCs, known to be negative regulators of advanced myelination,^[^ ^30^, ^31^, ^32^ ^]^ persisted as strongly upregulated in P10 and 2‐month‐old cKO nerves. According to a strong inhibitory impact on myelination, *Egr2*, *Myrf*, and *Myrfl* expression appeared downregulated in cKO nerves compared to age‐matched control nerves (Figure 4e). Notably, immunofluorescence analysis showed persistent SOX2 staining in 2‐month‐old sciatic nerve sections, but not in control ones (Figure S4a, Supporting Information).

At P10 an increased percentage of c‐Jun‐positive nuclei was also present in cKO compared to controls (Figure 4f), pointing to a fraction of SCs undergoing de‐differentiation.^[^ ^33^ ^]^ Indeed, mutant nerves displayed a low percentage of preserved myelinated axons at P10, but classical features related to myelin degradation were hardly detectable either at P10, where myelination is not yet completed, as well as at 2 months of age, when instead myelinated axons already disappeared in cKO samples (Figure 2c,d). To investigate how myelin was cleared between the stage of P10 and 2 months of age, we assessed nerve ultrastructure by TEM analysis at an intermediate timepoint. At P21, myelin membranes undergoing degradation were widely detectable (Figure S4b, Supporting Information). Remarkably, at this stage we observed myelin debris undergoing exocytosis in demyelinating SCs, together with macrophages containing myelin membranes undergoing terminal degradation, which contacted axons underneath SC basal lamina (Figure S4c, Supporting Information), pointing at exocytosis and macrophage phagocytosis as main responsible for myelin clearance.^[^ ^34^ ^]^

**Figure 5:**
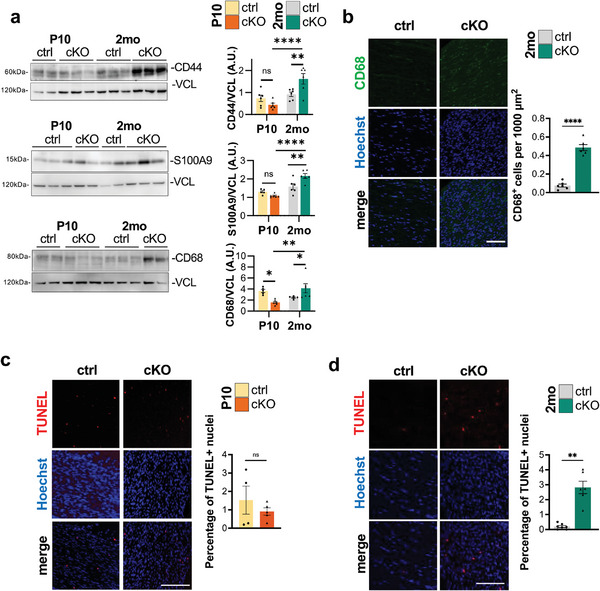
a) Representative western blot images and the relative densitometric quantifications of the immune‐related markers CD44, S100A9, and CD68 on total sciatic nerves protein extracts of P10 and 2‐month‐old control and *Becn1* cKO mice. Vinculin (VCL) was used as loading control (*n* = 5–6 mice each group; ns, not significant; ^*^ *P* < 0.05; ^**^ *P* < 0.01; ^****^ *P* < 0.0001; 2‐way ANOVA with Holm‐Šídák's multiple comparisons test). b) Representative confocal immunofluorescence images and the relative quantification of the number of CD68‐positive cells (green) per area, on longitudinal cryosection of sciatic nerves from 2‐month‐old control and *Becn1* cKO mice. Nuclei were counterstained with Hoechst (blue). Scale bar = 50 µm (*n* = 6 nerves sampled from 3 mice each group; ^****^ *P* < 0.0001; unpaired Student's *t*‐test). c,d) Representative images of TUNEL analysis on cryosections from P10 (c) e 2‐month‐old (d) control and *Becn1* cKO mice and relative quantification of the percentage of apoptotic nuclei (red). Nuclei were counterstained with Hoechst (blue). Scale bar = 50 µm (P10, *n* = 4 nerves sampled from 2 mice each group; 2 mo, *n* = 6 nerves sampled from 3 mice each group, ^**^ *P* < 0.01; Welch's t test). 2 mo, 2‐month‐old; ctrl, control; A.U., arbitrary units.

Moreover, looking at the GSEA (Figure 4a,b), a great upregulation in genes related to immune response specifically appeared in 2‐month‐old *Becn1* cKO samples, while were absent in P10 *Becn1* cKO ones, suggesting a progressive immune infiltration in adult *Becn1* cKO mice. We further validated the presence of neutrophil and macrophage markers in sciatic nerve protein extracts, unraveling a significant increase in CD44, S100A9, and CD68 protein levels in 2‐month‐old *Becn1* cKO mice compared to age‐matched controls as well as to P10 samples (**Figure** **5**a). Immunofluorescence analysis further confirmed an increase of CD68‐positive cells in 2‐month‐old *Becn1* cKO sciatic nerves (Figure 5b). To assess whether the observed immune response was triggered by SCs undergoing cell death, we performed TUNEL on cryosections of sciatic nerves from P10 and 2‐month‐old mice. While analysis at P10 did not highlight increased apoptosis in cKO nerves (Figure 5c), apoptotic nuclei were significantly increased in adult cKO nerves compared to controls (Figure 5d). These data point to the recruitment of inflammatory cells to the peripheral nerves in later stages, in the presence of progressively altered SCs due to Beclin 1 lack. On the other hand, the downregulated genes in *Becn1* cKO samples highlighted impairments in nervous system development, synaptic signaling, neuronal morphogenesis, neurite outgrowth, and muscular contraction, underlying how the observed phenotypic defects are reflected also by transcriptional responses in adult mice, and how the presence of alterations in SCs in adults starts impacting also the neuronal counterpart (Figure 4a,b).

### 2.6 *Becn1* cKO Mice Developed Motor Neuron Impairments Leading to Motor Dysfunction

**Figure 6:**
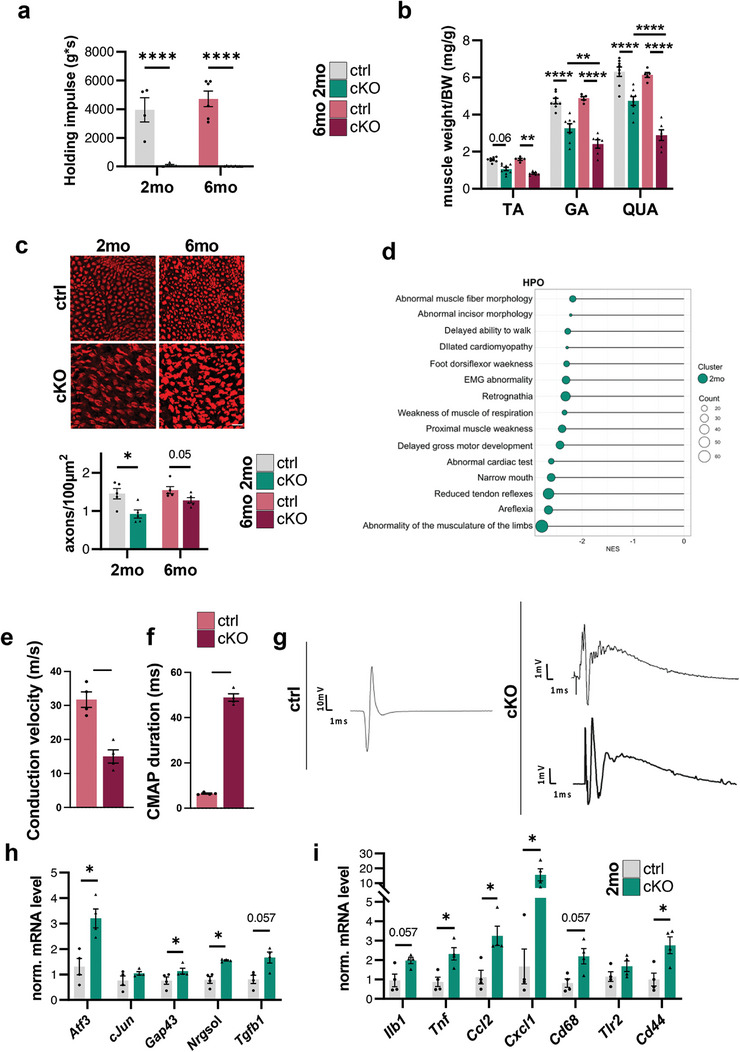
a) Recorded hanging time performances normalized per body weight (Holding impulse, g^*^s), as determined by the four‐limb hanging test in 2‐month‐old and 6‐month‐old control and *Becn1* cKO mice (*n* = 5–9 mice each group; ^****^ *P* < 0.0001; 2‐way ANOVA with Holm‐Šídák's multiple comparisons test). b) Tibialis anterior (TA), gastrocnemius (GA) and quadriceps (QUA) muscle weight normalized on body weight (BW) (mg/g) of 2‐month‐old and 6‐month‐old control and *Becn1* cKO mice (*n* = 5–8 mice each group; ^**^ *P* < 0.01; ^****^ *P* < 0.0001; 2‐way ANOVA with Holm‐Šídák's multiple comparisons test). c) Representative confocal immunofluorescence images for peripherin (red) and relative quantification of the number of axons per 100 µm^2^ of area manually counted on entire transversal cryosection of sciatic nerves from 2‐month‐old and 6‐month‐old control and *Becn1* cKO mice. Scale bar = 20 µm (*n* = 5 mice each group; ^*^ *P* < 0.05; Mann‐Whitney U test). d) Dot plot summarizing the most significant negatively enriched HPO terms (top 15) for DEGs in 2‐month‐old *Becn1* cKO mice. The size of the circle corresponds to the count of genes enriched in each gene sets. e,f) Recorded nerve conduction velocity (NCV) and compound muscle action potential (CMAP) duration measured in 5‐month‐old control and *Becn1* cKO mice (n = 4 mice each genotype; ^*^ *P* < 0.05; Mann‐Whitney U test). g) Representative CMAP curves recorded from 5‐month‐old mice control and *Becn1* cKO mice. Recording scales are provided. h,i) RT‐qPCR for axon damage‐ (h) and immune infiltrate‐related (i) transcripts in spinal cord mRNA extracts of 2‐month‐old control and *Becn1* cKO mice (*n* = 4 mice each group; ^*^ *P* < 0.05; Mann‐Whitney U test). NES, Normalized Enrichment Score; 2 mo, 2‐month‐old; 6 mo, 6‐month‐old; ctrl, control.

Since the lack of Beclin 1 in SCs led to the development of a remarkable neuropathic phenotype, including involuntary tremors and walking difficulties, we further investigated the involvement of the motor compartment. First, to have a quantitative readout of the motor function and behavior, we performed the four‐limb hanging test on 2‐ and 6‐month‐old mice, observing the complete failure of *Becn1* cKO mice in hanging on an upside‐down reversed grid with respect to control littermates (**Figure** **6**a). Likewise, cKO mice displayed a dramatically reduced performance on the rotarod test, when compared to age‐matched controls (Figure S6a, Supporting Information). Moreover, we found that, along with body weight decrease (Figure 1d), 2‐ and 6‐month‐old *Becn1* cKO mice underwent a significant and massive loss of skeletal muscle mass (normalized on body weight) when compared to control animals (Figure 6b), compatible with denervation‐induced muscle atrophy. Indeed, to understand if the SC defects were also impinging on motor neurons, we counted the number of motor axons per area in transversal sciatic nerve sections of 2‐ and 6‐month‐old mice. At both ages we described a decrease in the number of motor axons per area in *Becn1* cKO mice when compared to control littermates (Figure 6c), suggesting that the lack of Beclin 1 in SCs could affect neuronal viability, as supported also by transcriptomic analysis in 2‐month‐old nerves (Figure 4a,b). Remarkably, GSEA with Human Phenotype Ontology also highlighted negatively enriched gene sets related to muscle and nervous system impairment in 2‐month‐old *Becn1* cKO samples (Figure 6d). Even more relevant in the functional context, electrophysiological analysis on 5‐month‐old mice reported a prominent slowdown in nerve conduction velocity (NCV) in cKO animals, compared to age‐matched controls (Figure 6e), reflecting the observed severe demyelination. The lower NCV in cKO nerves was accompanied by compound muscle action potentials displaying a wider duration (Figure 6f) and polyphasic curves of lower intensity in terms of recording scales (Figure 6g), consistent with chronic denervation and also matching with secondary axonal degeneration. To investigate the impact of such defective neurotransmission on target tissues, we also monitored changes in neuromuscular junction (NMJ) innervation in tibialis anterior longitudinal cryosections from 2‐month‐old mice. Notably, a significant increase in the percentage of NMJs that were only partially innervated was detected in *Becn1* cKO mice when compared to controls (Figure S6b,c, Supporting Information), while the analysis of post‐synaptic acetylcholine receptor clusters’ fragmentation did not highlight any significant difference (Figure S6d–f, Supporting Information). To better understand if motor neurons were subjected to injury‐like stress and/or degeneration, we also analyzed the levels of some key transcripts in spinal cord mRNA extracts from 2‐month‐old animals. Indeed, transcripts typically responsive to injury, including *Atf3* and *Gap43* ^[^ ^35^, ^36^ ^]^ were upregulated in cKO samples compared to controls (Figure 6h). As well, soluble neuregulin (*Nrg*sol) and *Tgfb1*, were shown to be upregulated in the spinal cord upon nerve injury and known to be able to orchestrate nerve repair mechanisms,^[^ ^37^, ^38^, ^39^ ^]^ resulted upregulated; while *cJun*, particularly involved in SC dedifferentiation^[^ ^33^ ^]^ was not altered in this compartment (Figure 6h). Since signs of inflammation were evident in nerves and commonly found in the spinal cord after nerve injury and in pathological conditions,^[^ ^40^ ^]^ we investigated their extension to the spinal cord. Interestingly we found increased expression of secreted cytokines and chemokines driving such response, including *Il1b*, *Tnf*, *Ccl2*, and *Cxcl1*, as well as upregulated transcripts of inflammatory cell markers, like *Cd68* and *Cd44* (Figure 6i). Altogether these data underline how profoundly Beclin 1 loss in SCs negatively affects motor neuron homeostasis, causing a remarkable injury‐like response in motor neuron cell bodies, with the consequent immune infiltration into the spinal cord.

### 2.7 Lack of Beclin 1 in SCs Led to Impaired Sensory Neuron Development and Function

**Figure 7:**
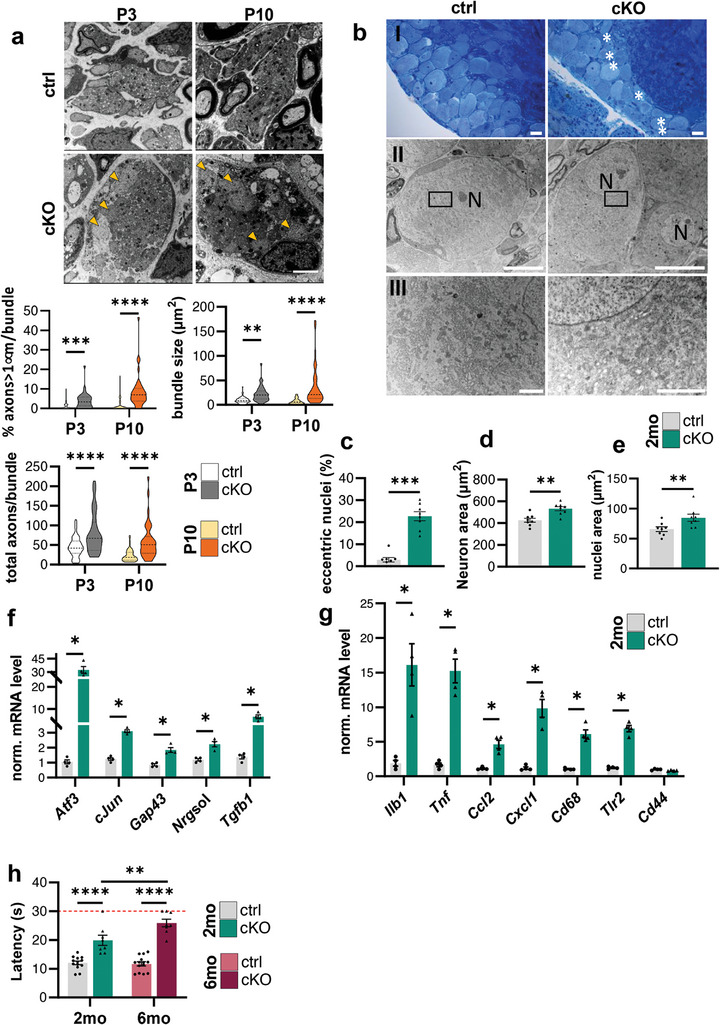
a) Representative high magnification transmission electron microscopy images of Remak bundles in developing sciatic nerve transversal sections from P3 and P10 control and *Becn1* cKO mice. Orange arrowheads indicate non‐sorted large caliber axons, scale bar = 2 µm. The relative violin plots show quantifications of respectively the percentage of axons with a diameter ≥ 1 µm per bundle, the bundles size (µm^2^), and the total number of axons per bundle, counted manually from images as in (a) (*n* = 39–70 bundles from 3 mice each group; ^**^ *P* < 0.01; ^***^ *P* < 0.001; ^****^ *P* < 0.0001; 2‐way ANOVA with Holm‐Šídák's multiple comparisons test). b, I) Representative images of toluidine blue‐stained semithin L1 ganglion sections from 2‐month‐old control and *Becn1* cKO mice; white asterisks indicate neurons with eccentric nuclei, scale bar = 20 µm. b, II‐III) Representative transmission electron microscopy images of L1 ganglion from 2‐month‐old control and *Becn1* cKO mice. Light yellow areas highlight Nissl bodies (III); scale bar = 10 µm (II), scale bar = 2 µm (III); N, nucleus. c–e) Quantification of the percentage of neurons with eccentric nucleus (c) and of the neuronal (d) and nuclei (e) area (µm^2^) and, as measured from images as in (b) (*n* = 8 mice each group; ^**^ *P* < 0.01; Mann‐Whitney U test). f, g) RT‐qPCR for neuron damage‐ (f) and immune infiltrate‐related (g) transcripts in DRGs mRNA extracts of 2‐month‐old control and *Becn1* cKO mice (*n* = 4 mice each group; ^*^ *P* < 0.05; Mann‐Whitney U test). h) Recorded latency time to react to constant high temperature (s), as determined by the hot plate test in 2‐month‐old and 6‐month‐old control and *Becn1* cKO mice. The red dotted line indicates the maximum time after which the experiment was stopped to avoid animal injury (*n* = 8–13 mice each group; ^**^ *P* < 0.01; ^****^ *P* < 0.0001; 2‐way ANOVA with Holm‐Šídák's multiple comparisons test). 2 mo, 2‐month‐old; 6 mo, 6‐month‐old; ctrl, control.

In mice, SC differentiation begins soon after birth with the formation of both myelinating and non‐myelinating SCs.^[^ ^41^ ^]^ Non‐myelinating SCs are associated with bundles of Remak fibers, mainly composed of afferent axons of sensory neurons.^[^ ^41^ ^]^ Considering the expression of *Mpz*‐Cre between embryonic days 13.5 and 14.5 in the progenitors of both myelinating and non‐myelinating SCs,^[^ ^25^, ^26^, ^27^ ^]^ we wanted to assess if the latter were affected by Beclin 1 ablation in our model. From late embryonic development to post‐natal day 10, large‐caliber axons are sorted by myelinating SCs, leaving small‐caliber axons associated with non‐myelinating SCs, in a process called radial sorting.^[^ ^5^, ^41^ ^]^ By analyzing axon bundles in sciatic nerve transversal TEM images 3 and 10 days after birth, we found that the lack of Beclin 1 in SCs altered the radial sorting process. In particular, we showed that, at both time points, the percentage of large caliber axons (with a diameter larger than 1 µm) retained in the bundles was significantly higher in *Becn1* cKO nerves compared to controls, and that the bundles’ area, as well as the total number of axons per bundles, were significantly increased in *Becn1* cKO nerves with respect to control littermates (**Figure** **7**a). These data support the hypothesis that when Beclin 1 is missing the radial sorting process does not occur properly or is, at least, delayed. To understand if sensory neurons were affected once development is completed, we analyzed neuronal cell bodies located in the dorsal root ganglia (DRGs). From a histological evaluation of toluidine blue‐stained L1 DRGs, we observed an increased presence of neurons with an eccentric nucleus in *Becn1* cKO samples compared to controls (Figure 7b‐I,II, white asterisks), an indicator of chromatolysis occurrence.^[^ ^42^, ^43^ ^]^ To better investigate this phenomenon, a quantification was carried out, confirming a significantly higher percentage of eccentric nuclei in *Becn1* cKO DRG compared to controls (Figure 7c), accompanied by a significant swelling of the perikaryon (Figure 7d) and nuclear enlargement (Figure 7e). In addition, *Becn1* cKO mice displayed dispersion of the rough endoplasmic reticulum, also called Nissl bodies, as shown in higher magnification TEM images (Figure 7b‐III, light yellow areas). These findings strengthened the hypothesis that the demyelinating neuropathy due to the lack of Beclin 1 in SCs caused damages not only to motor fibers but also to sensory axons, capable of inducing a chromatolytic response in DRG cell bodies, reflected by all these typical histological hallmarks we found in *Becn1* cKO DRGs.^[^ ^44^ ^]^ This injury‐like response was well mirrored also by the analysis of key transcripts from thoracic DRGs mRNA extracts. Indeed, as previously shown also in the spinal cord, we found enhanced expression of genes commonly involved in injury response,^[^ ^45^, ^46^ ^]^ such as *Atf3*, *cJun*, *Gap43*, *Nrgsol*, and *Tgfb1* (Figure 7f), accompanied by increased expression of secreted cytokines and chemokines, including *Ilb1*, *Tnf*, *Ccl2*, and *Cxcl1*, as well as upregulated transcripts of inflammatory cells like *Cd68* and *Tlr2* (Figure 7g). Immunofluorescence analysis of immune cell markers further confirmed increased positivity for CD68‐ and Iba1‐positive cells in cKO DRG sections, when compared to controls (Figure S5, Supporting Information). Finally, we performed a behavioral test for sensory functions, the hot plate test, on 2‐ and 6‐month‐old mice. *Becn1* cKO mice displayed a significantly increased latency to manifest nocifensive behavior with respect to control littermates, and interestingly the delay in the response increased even more in 6‐month‐old *Becn1* cKO mice when compared to 2‐month‐old *Becn1* cKO mice, demonstrating a progressive worsening of the phenotype in functional impairment of sensory fibers (Figure 7h).

### 2.8 Beclin 1 Results as a Central Hub in SC Maturation, Integrating Diverse Signaling Pathways

In order to have deeper insights into the mechanisms underlying the molecular, histological, and functional neuropathic phenotype of *Becn1* cKO mice, we analyzed dynamic transcriptomic changes occurring over time during the acquisition of this phenotype. Time‐series analysis identified subgroups of correlated genes with specific expression patterns between the two considered time points (Figure S3f, Supporting Information). To highlight classes of transcripts that should be up‐regulated during normal development but were affected in *Becn1* cKO mice, guiding our subsequent mechanistic analysis on deregulated signals in 2‐month‐old median nerve protein extracts, we focused on two defined clusters of genes characterized by a down‐regulated and flat pattern of expression in cKO over time and a progressive increase of expression in controls (Figure S3f,g, Supporting Information).

**Figure 8:**
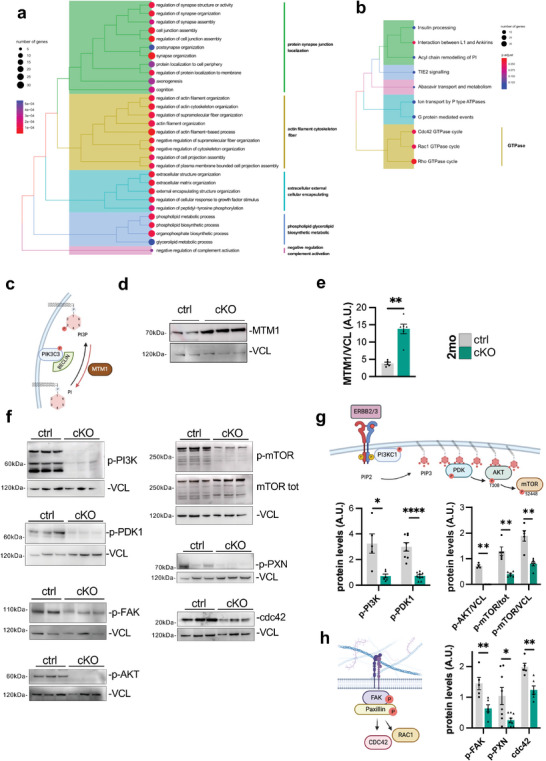
a,b) Treeplot summarizing over‐representing analysis on GO‐BP (a) and Reactome (b) for selected cluster of genes with correlated pattern of expression by time‐series analysis. Node colors by p.adjusted and size by number of genes enriched in each term. c) Schematic representation of Beclin 1 role in phospho‐inositol (PI) metabolism. d,e) Representative western blot images (d) and relative densitometric quantifications (e) for myotubularin 1 (MTM1) on total median nerve protein extracts of 2‐month‐old control and *Becn1* cKO mice. Vinculin (VCL) was used as a loading control (*n* = 5–6 mice each group; ^**^ *P* < 0.01; Mann‐Whitney U test). f,h) Representative western blot images (f) and the relative densitometric quantifications g,h) for PI3K/AKT/mTOR axis proteins (g) [phosphorylated class I phosphoinositide 3‐kinase p85/p55 (p‐PI3K), phospho‐PDK1 (p‐PDK1), phospho‐AKT (p‐AKT), phospho‐mTOR (p‐mTOR) and total mTOR (mTOR tot)], and for ECM‐driven signaling proteins (h) [phospho‐FAK (p‐FAK), phospho‐paxillin (p‐PXN), cdc42], on total median nerves protein extracts of 2‐month‐old control and *Becn1* cKO mice. Vinculin (VCL) was used as loading control (*n* = 5–9 mice each group; ^*^ *P* < 0.05; ^**^ *P* < 0.01; Mann‐Whitney U test). 2 mo, 2‐month‐old; ctrl, control; A.U., arbitrary units. Schematics in (c,g,h) were created with BioRender.com.

Over‐representation analysis (**Figure** **8**a,b) showed that phospholipid biosynthetic and metabolic processes are affected in cKO nerves (Figure 8a). Indeed, Beclin 1 is a major interactor of PIK3C3 (or Vps34, Figure 8c), a kinase regulated by and involved in the production of PI3P,^[^ ^47^ ^]^ which in turn is crucial for the regulation of many intracellular pathways including membrane trafficking^[^ ^48^ ^]^ and appeared downregulated already at P10 when analyzed by immunofluorescence (Figure S7, Supporting Information). Interestingly, we observed that the protein levels of the PI3P phosphatase myotubularin 1 (MTM1), which instead depletes the PI3P pool, but also directly targets the PIK3C3^[^ ^49^ ^]^ (Figure 8c), were significantly higher in *Becn1* cKO mice with respect to controls (Figure 8d,e). We investigated other SC‐relevant kinases involved in phosphoinositides’ biosynthesis and found that class I PI3K regulatory phosphorylations were strongly decreased in *Becn1* cKO mice with respect to controls (Figure 8f,g). Coherently with PI3K dephosphorylation in *Becn1* cKO nerves, we found that also the downstream PI3K/AKT/mTOR axis was inactivated in *Becn1* cKO mice, with a strong and significant decrease in *i)* 3‐phosphoinositide‐dependent protein kinase‐1 (PDK1) phosphorylation, normally driven by the presence of PIP3,^[^ ^50^ ^]^ *ii)* phosphorylation level of the PDK1 target Thr308 on AKT, and *iii)* phospho‐mTOR levels (Figure 8f,g).

Concurrently, as also emerged from GO‐Biological Processes (Figure 8a), the signaling axis that mediates the response to extracellular ligands and extracellular matrix signals^[^ ^51^ ^]^ appeared to be dysregulated, as confirmed in *Becn1* cKO nerves by the significant decrease of Focal Adhesion Kinase (FAK) and Paxillin phosphorylation, as well as of the protein levels of cdc42 (Figure 8f,h), a well‐established component of the Rho GTPase family, involved in SC development and myelination.^[^ ^52^ ^]^ Interestingly cdc42 and its closely related Rho and Rac1 GTPases, resulted as the three most deregulated drivers of transcript classes during development in *Becn1* cKO nerves, according to the Reactome enrichment (Figure 8b). Altogether these data highlighted how Beclin 1 loss in SCs impinges on diverse signaling pathways, on the one hand governed by the extracellular matrix, on the other hand, more directly involved with the function of Beclin 1 interactors (i.e., PIK3C3), and in integrating axonal signals.

## 3 Discussion

Endosomal trafficking and autophagy play a key role in nervous system development and homeostasis, as well as in SCs differentiation and injury response.^[^ ^53^, ^54^ ^]^ Additionally, it is known that autophagy is deregulated in neurodegeneration, although past studies were mainly focused on the neuronal compartment, rather than on glial cells.^[^ ^55^, ^56^ ^]^ In this framework, we focused our studies on investigating the role that Beclin 1 exerts in SCs, as a key regulator of autophagy induction and membrane trafficking. Toward this aim, we successfully generated and characterized a new mouse model in which *Becn1* gene is specifically knocked out in the myelinating glia of the PNS since embryonic stages. We found that *Becn1* cKO mice develop a severe recessive, and progressive dysmyelinating peripheral neuropathy, characterized by extensive involuntary tremors, motor and sensory impairments, late paresis of posterior legs, massive muscle atrophy, and body weight loss.

The absence of Beclin 1 induced remarkable defects within SCs, such as the presence of an enlarged cytoplasm characterized by the accretion of copious membrane vesicles, already detectable since few days after birth. This evidence, together with the parallel accumulation of autophagic markers, in particular of p62, in *Becn1* cKO nerves, is consistent with an altered degradation of cytoplasmic materials together with dysregulation of membrane trafficking. Indeed, a defect in the later steps of autophagy degradative action, as well as in endosomal maturation, clearly resulted upon in vivo *Becn1* deregulation in other cell types, including hippocampal neurons and adipocytes.^[^ ^17^, ^57^ ^]^

Since proper myelination requires both the correct delivery of membranes and myelin proteins to the plasma membrane,^[^ ^58^, ^59^ ^]^ as well as the autophagic removal of cytoplasm portions for myelin compaction,^[^ ^9^ ^]^ the impairment in membrane trafficking is expected to contribute to the strong demyelination observed in *Becn1* cKO animals. Accordingly, while it is reported that autophagy‐ (*Atg7*‐) deficient SCs maintain a thickened abaxonal cytoplasm in adult stages but do properly carry out myelination,^[^ ^9^, ^54^ ^]^ a primary impairment in endosomal trafficking and maturation was sufficient to compromise myelination in SC‐specific Vps34 or dynamin 2 knockout mice.^[^ ^60^, ^61^ ^]^ In the first case the deletion of the (Beclin 1 interactor) enzymatic core of the Class III PI3K complex, responsible for PI3P production and directly contributing to endosomal sorting,^[^ ^47^ ^]^ resulted in a marked hypomyelination and affected radial sorting, accompanied by SC autophagic impairment, accumulation of cytoplasmic vacuoles, and SC hyperproliferation.^[^ ^60^ ^]^ Complementarily, the ablation of dynamin 2, a large GTPase with major roles in membrane fission and endocytosis,^[^ ^62^, ^63^, ^64^ ^]^ determined defective myelination and radial sorting, delayed SC differentiation, and mitotic processes together with ablated myelin‐related protein levels.^[^ ^61^ ^]^ These models strongly underpin a role for altered endosomal trafficking also in radial sorting impairment, as observed in our *Becn1* cKO model.

In line with the effects on SC maturation and proliferation observed in the mentioned models, our transcriptomic analysis pointed to enhanced gene expression related to the control of cell cycle and pro‐mitotic events at both P10 and 2 months, as also confirmed by Ki‐67 and c‐Jun‐immunostaining on sections at P10, underlying either a delay in SC differentiation or a dedifferentiation program initiated by affected SCs. Indeed, transcriptomic analysis also highlighted a persistent upregulation of transcription factors, including *Tfap2a, Pou3f1, and Pou3f2*, as well as *Sox2*, at p10 and 2 months of age. Such deregulation is likely responsible for the observed hypomyelination. Nonetheless, myelination seems to occur in cKO at early stages (P3 and P10), but the percentage of myelinated axons in mutant adults drops to almost undetectable levels.

Most of the knowledge regarding key events in demyelinating SCs was acquired by studying injury‐induced Wallerian degeneration, whose features – including increased SC proliferation, c‐Jun upregulation, downregulation of myelin‐related genes and boosted lysosomal compartment – are shared also by SCs undergoing demyelination in the absence of acute nerve damage.^[^ ^65^, ^66^ ^]^ The observed increased proliferation, decreased expression of pro‐myelinating genes, and almost complete abatement of myelin‐related protein levels, together with increased apoptosis, strongly correlate with an injury‐related response occurring in *Becn1* cKO SCs.

Other signs point to the presence of an injury‐like condition. Indeed, according to GSEA, while at P10 the most upregulated genes can be ascribed to a cell‐autonomous scenario (GO terms: cell cycle process, cell division, chromosome organization, and others), transcriptomic analysis revealed defects spreading at a systemic level in 2‐month‐old cKO nerves, where enhanced immune response and defective processes related to neuronal growth and neurotransmission are identified among deregulated gene sets. Remarkably, the levels of immune‐related proteins that were not significantly different in cKO nerves at P10, became significantly increased at 2 months of age, making evident the local recruitment of immune cells. This is again a feature shared by injury‐related demyelination as well as by acquired, hereditary or genetically induced demyelination, where the loss of contact between axons and defective SCs induces the secretion of cytokines able to recruit and activate CD68‐ and CD44‐positive macrophages, as well as S100A9‐positive neutrophils.^[^ ^65^, ^67^, ^68^, ^69^ ^]^ Of note, CD44 was shown to mark not only immune cells but also demyelinating Schwann cells upon nerve injury and to have a role in mediating neuregulin signaling through ErbB receptors, being essential in establishing axon‐SC contacts.^[^ ^70^, ^71^ ^]^

A loss of contact between SCs and axons is expected in demyelinated *Becn1* cKO nerves, as appreciated by TEM analysis pointing at naked axons, and as deduced by the upregulation of genes including *Atf3*, *Gap43*, and *Nrg1* and *cJun* in cKO DRG and spinal cord in mice, as previously reported upon experimental nerve injury.^[^ ^46^, ^72^ ^]^ In keeping with the manifested motor and sensory impairments and the transcriptomic deregulation of gene sets related to neurotransmission and neurodevelopment in *Becn1* cKO mice, the presence of secondary axonal loss was further confirmed in *Becn1*cKO sciatic nerves since 2 months of age, also impacting on NMJ innervation. This is in line with clinical impairment described in different forms of demyelinating neuropathies and ascribed to secondary axonal damage, rather than to reduced conduction velocity caused by the sole loss of myelin insulation.^[^ ^8^, ^73^ ^]^ Several mechanisms might underlie such degeneration, including heightened energy demand and altered ion gradient due to voltage‐gated channels mislocalization,^[^ ^74^ ^]^ a drop of essential metabolic intermediates^[^ ^75^ ^]^ or neuroinflammation triggered by SC defects.^[^ ^76^ ^]^ In *Becn1* cKO mice, such signs of neuroinflammation reached even the DRG and the spinal cord in terms of upregulation of cytokines including *Tgfb1*, *Tnf*, *Ilb1*, *Ccl2*, and *Cxcl1* and immune cell markers *Cd68* and *Cd44*, accounting once more for a systemic spread of SC defect influence.

Therefore, in sum, this work showed that SC‐specific ablation of Beclin 1 in mice induced a demyelinating neuropathic phenotype with reduced NCV, a motor‐sensory impairment and axonal degeneration characterized by local immune cell recruitment in sciatic nerves, as well as motor and sensory neuron cell body response and signs of neuroinflammation and neurodegeneration in adult mice.

Such aspects strongly correlate with congenital demyelinating Charcot‐Marie‐Tooth‐(CMT) diseases, including the autosomal dominant CMT1, the autosomal recessive CMT4, and the X‐linked types, that display a progressive impairment in the maintenance of myelin sheaths and of axon‐glial contacts with a characteristic reduction in motor conduction velocity and other shared features including muscle weakness and atrophy, sensory loss and foot deformities.^[^ ^77^, ^78^ ^]^ The hypomyelination observed in *Becn1* cKO nerves, resulting in a reduced number of myelinated axons, as well as the consequent impact on the significantly reduced NCV is compatible with features of demyelinating CMT diseases. The observed muscle weakness and sensory loss further support such parallel. Of note, SC dedifferentiation and the ensuing increased proliferation appear to be typical features of nerve fiber damage further associated with several forms of demyelinating CMT.^[^ ^79^, ^80^, ^81^, ^82^ ^]^ Not only the SC‐autonomous aspects are similar, but also secondary axonal degeneration, frequently reported for CMT diseases,^[^ ^83^ ^]^ is present in *Becn1* cKO nerves. Instead, immune cell recruitment and inflammation, although widely described in animal models of CMT diseases^[^ ^80^, ^84^ ^]^ seems to be less reported in histological characterizations of patients’ samples, likely also reflecting an increasingly less frequent resort to nerve biopsy in clinical practice.^[^ ^85^ ^]^

Going back to SC defects and focusing on the signalling pathways involved, in our work SC maturation toward both the myelinating and the unmyelinating Remak phenotype appears altered during development in *Becn1* cKO mice. The differentiation process requires SCs to properly integrate signals deriving by both their secreted basal lamina and the growing axons, in turn initiating vital intracellular signaling cascades.^[^ ^3^, ^25^, ^51^, ^86^ ^]^ In our investigations Beclin 1 emerges as a major hub in this integrating action, since, upon its lack, different pathways were strongly deregulated: *i)* the PI3K/AKT/mTOR axis, that in turn mediates adaxonal neuregulin/ERBB3 signal cascade^[^ ^87^ ^]^ but also abaxonal integrin β4 signalings,^[^ ^88^ ^]^ ii) FAK and paxillin phosphorylation, mostly transducing extracellular matrix‐driven signals.^[^ ^89^, ^90^ ^]^ Both these deregulated pathways could impinge on the downstream target cdc42, known to be crucial in SC development and myelination.^[^ ^91^, ^92^, ^93^ ^]^

Of note, Beclin 1 is the major interactor of PIK3C3 (or Vps34), a kinase directly involved in the production of PI3P. The lower reactivity for PI3P detected in P10 sciatic nerve sections together with the increased protein level of MTM1, which depletes the PI3P pool but also directly targets the PIK3C3,^[^ ^49^, ^50^ ^]^ corroborate the presence of a depletion of PI3P pools in cKO nerves. Our data suggest the presence of an unbalance in different PI species, known to govern membrane trafficking^[^ ^48^ ^]^ and show a global alteration that impacts on the activation state of other phosphoinositide‐related signaling players, such as class I PI3K, found to be remarkably inactivated in 2‐month‐old *Becn1* cKO samples.

Therefore, we believe that our model supports the recognized importance of regulated phosphatidyl‐inositide production in myelination and neuropathies. Further evidence comes from models including the SC‐specific knockout mice for proteins affecting phosphatidyl‐inositides synthesis and balance, including PI3K,^[^ ^60^ ^]^ PI4K subunits (PI4KB and PI4KA),^[^ ^94^, ^95^ ^]^ FIG4,^[^ ^96^, ^97^ ^]^ but also the MTMR family of phosphatases,^[^ ^98^ ^]^ all resulting in strongly affected myelination, often accompanied by defects at the level of Remak bundles. Besides their relevance in basic knowledge about myelination, as modulated by membrane lipid‐content, some of these mice are also established disease models for human demyelinating diseases and key tools for testing novel therapeutical strategies.^[^ ^78^, ^99^ ^]^ Likewise, with this work, Beclin 1 emerges with a major role in SC homeostasis and as a candidate target in the modulation of PI3P‐associated signals, typically altered in demyelinating neuropathies.

## 4 Experimental Section

### Mouse Maintenance and Genotyping

All mice were housed in a controlled environment with 12 h light/12 h dark cycle, a temperature of 23° C, and with free access to water and standard chow (Mucedola Srl, 4RF25). *Becn1* cKO mice were generated by crossing *Becn1^fl/fl^* mice (Monterotondo Mouse Clinic) with mice bearing the Cre recombinase under the control of the *Mpz* promoter (kindly provided by Dr. Stefano Previtali, IRCCS San Raffaele Scientific Institute, Milano, Italy). After line expansion, the crossing between *Becn1* ^fl/+^:*Mpz*‐Cre and *Becn1* ^fl/fl^ gives birth to all the three genotypes investigated in the present study: control animals (*Becn1* ^fl/fl^ or *Becn1* ^fl/+^ mice), heterozygous animals (*Becn1* cHet, *Becn1* ^fl/+^:*Mpz*‐Cre mice), and conditional knockout animals (*Becn1* cKO, *Becn1* ^fl/fl^:*Mpz*‐Cre mice). Genotypes were assessed by PCR analysis performed on DNA extracted from ear biopsies (primers are listed in Table S1, Supporting Information). When experiments involved pups 10 days after birth (P10), PCR was also performed to discriminate mouse gender.^[^ ^100^ ^]^ Both male and female animals were sacrificed by cervical dislocation, or by decapitation in the case of pups, and the tissues of interest were immediately dissected and fixed, included or frozen in nitrogen vapors. Skeletal muscles were weighted just after dissection, prior to the freezing by isopentane immersion in nitrogen vapors. In order to avoid undesired effects due to circadian variations, all the mice were sacrificed in the morning. The adopted procedures were approved by the Ethics Committee of the University of Padova and carried out according to all pertinent Italian laws (OPBA, n. 285/2018 and n. 501/2023).

### Behavioral Tests

The *Four Limb Hanging Test* was performed according to the TREAT‐NMD standard operating procedures (DMD_M.2.1.005; https://treat‐nmd.org). Briefly, mice were placed on a grid, which was kept upside down 40 cm above an empty cage filled with bedding. The hanging time, that is, the time until the animal fell, was recorded, with a fixed upper limit of 180 s. Each mouse was tested twice on two consecutive days. Holding impulse was obtained by multiplying the hanging time for the respective mouse body weight, then the maximum holding time was used for statistical analysis. The *Rotarod test* was performed as follows. Mice were trained a week before the test by placing them on a constant rotation speed (4 rpm) on the rotarod (Ugo Basile, 47650). On the day of the test, mice were placed on the rotarod at 4 rpm. As soon as all mice were placed, the rotarod was set to accelerate up to 40 rpm in 300 s. Each mouse was tested three times with at least a 10‐min‐long interval among trials. 300 s was the maximum time achievable. Data was plotted considering the best performance obtained among the three trials. The *Hot plate test* was performed following the manufacturer's instructions (Ugo Basile S.R.L). Briefly, the temperature was maintained constant at 52 °C, the animals were placed on the pre‐warmed plate until the appearance of a nocifensive behavior (mainly the forelimb licking). The response latency to manifest a nocifensive behavior was recorded, with a maximum threshold of 30 s, after which the experiment was stopped to avoid animal injury. The experiment was performed twice, at 7 days of distance, then the average latency (s) was considered for the statistical analysis.

### Electrophysiological Measurements

Upon general anesthesia, the sciatic nerve was exposed through an incision in the trochanteric region, removing the skin and the biceps femoris muscle. Curved forceps were used to gently disrupt the connective tissue beneath the sciatic nerve, allowing the placement of a small piece of parafilm (1 cm width) underneath it. A pair of stimulating needle electrodes (Grass, USA) were carefully positioned on the exposed nerve, aided by a mechanical micromanipulator (MM33, FST, Germany). A pair of electromyography needle electrodes (Grass, USA) was used for electromyography recording of gastrocnemius muscle fibre activity. The recording needle electrode was inserted halfway into the gastrocnemius muscle, while the indifferent needle electrode was inserted in the distal tendon of the muscle. CMAPs were recorded following supramaximal stimulation of the sciatic nerve at 0.5 Hz (0.4 ms stimulus duration) using a stimulator (S88, Grass, USA) via a stimulus isolation unit (SIU5, Grass, USA) in a capacitance coupling mode. To reach supramaximal stimuli (5–15 mV for controls, up to 50 mV after nerve damage), the sciatic nerve was stimulated with increasingly intense stimuli until the CMAP value ceased to increase. Recorded signals were amplified by an extracellular amplifier (P6 Grass, USA), digitized using a digital A/C interface (National Instruments, USA), and then fed to a computer for both on‐line visualization and off‐line analysis using appropriate software (WinEDR, Strathclyde University; pClamp, Axon, USA). Stored data were analyzed off‐line using pClamp software (Axon, USA). Nerve conduction velocity was calculated by stimulating the sciatic nerve in two distinct locations and measuring a) the differences in latency between the two CMAPs evoked, and b) the distance between the stimulating sites. Latency was defined as the time between the deflection of the path induced by the electrical stimulation of the nerve and the beginning of the positive/negative wave of the CMAP. The 1‐cm width of the parafilm served as a reference for the stimulation points. The conduction velocity was expressed in m/sec. 4 mice per genotype were analyzed.

### Histology

For sciatic nerve histology, a central segment of sciatic nerve, soon after dissection, was fixed with 2.5% glutaraldehyde + 2% PFA in 0.1 M sodium cacodylate buffer pH 7.4 overnight at 4 °C. The sample was then postfixed with 1% osmium tetroxide in 0.1 m sodium cacodylate buffer for 2 h at 4 °C. After three water washes, samples were dehydrated in a graded ethanol series and embedded in an epoxy resin (Sigma–Aldrich). Ultrathin sections (60–70 nm) were obtained with an Ultratome Leica Ultracut EM UC7 ultramicrotome, counterstained with uranyl acetate and lead citrate, and viewed with a Tecnai G^2^ (FEI) transmission electron microscope operating at 100 kV. Images were captured with a Veleta (Olympus Soft Imaging System) digital camera and then analyzed by exploiting Fiji software. From the same samples, semi‐fine sections (1 µm) were also cut and stained with a 1% toluidine blue solution. The obtained slides were then mounted with immersion oil and representative images were taken with a light microscope (Leica 5000B).

For high‐resolution light and transmission electron microscopy of DRGs, samples were first fixed in 2.5% glutaraldehyde in 0.1 m phosphate buffer (pH 7.4) for at least 2 h at 4 °C and then were post‐fixed with 2% osmium tetroxide for 2 h and dehydrated in ethanol from 30% to 100% (5 min each passage). After two passages of 7 min in propylene oxide and 1 h in a 1:1 mixture of propylene oxide and Glauerts' mixture of resins, samples were embedded in Glauerts’ mixture of resins (made of equal parts of Araldite M and the Araldite Harter, HY 964, Sigma Aldrich). In the resin mixture, 0.5% of the plasticizer dibutyl phthalate (Sigma Aldrich) was added. For the final step, 2% of accelerator 964 was added to the resin in order to promote the polymerization of the embedding mixture, at 60 °C. For high‐resolution light microscopic analysis transverse semithin sections (2.5 µm thick) were obtained using an ultramicrotome (Ultracut UCT, Leica), and stained with 1% toluidine blue and 2% borate in distilled water. To evaluate DRG neurons area, area of nuclei, and percentage of neurons with eccentric nuclei, a total of 300 neurons for each experimental group was considered; quantification was performed on the toluidine blue‐stained semithin sections using a magnification of 100x. A DM4000B microscope equipped with a DFC320 digital camera was used for section analysis. On the same samples, ultrathin sections (70 nm) were obtained and were examined under a transmission electron microscope (JEOL, JEM‐1010) equipped with a Mega‐View‐III digital camera and a Soft‐Imaging‐System (SIS, Germany) for computerized acquisition of the images.

### Immunofluorescence

A segment of the sciatic nerve, soon after dissection, was included in OCT and then frozen in liquid nitrogen vapors. For autophagic markers, myelin and axons’ evaluations, longitudinal or transversal (10‐µm‐thick) sciatic nerve cryosections were permeabilized and fixed in 1:1 methanol‐acetone solution for 10 min at −20° C, washed, and blocked in 10% goat serum in phosphate‐buffered saline (PBS, NaCl 80 mg mL^−1^, KCl 2 mg mL^−1^, Na_2_HPO_4_ ·7H_2_O 11.5 mg mL^−1^, KH_2_PO_4_ 2 mg mL^−1^ in ddH_2_O) for 1 h at room temperature. For proliferation experiments, myelin and immune cell staining, longitudinal (10‐µm‐thick) sciatic nerve cryosections were post‐fixed in 4% PFA in PBS, permeabilized and blocked for 2 h in a solution of 5% IgG free bovine serum albumin (BSA Sigma‐Aldrich, A7030), 0.5% Triton X‐100 (Sigma‐Aldrich, T9284) in PBS at room temperature. Then, an anti‐mouse Fab fragment antibody (1:25) was incubated for 30 min to block endogenous IgG and washed. All slides were then incubated overnight at 4 °C with the respective primary antibodies: guinea pig anti‐p62 (1:100, Progen, GP62‐C); rat anti‐LAMP1 (1:100, DSHB, 1D4B); rabbit anti‐peripherin (1:100, Novus Biologicals, NB300‐137); rat anti‐MBP (1:100, Abcam, ab7349); rabbit anti‐Ki67 (1:50, Novus Biologicals, NB600‐1252); mouse anti‐SOX10 (1:50, Sigma–Aldrich, AMAB91297); rabbit anti‐MPZ (1:300, Abcam, ab31851); rat anti‐CD68 (1:100,Thermo Fisher Scientific, 14‐0681‐82); rabbit anti‐SOX2 (1:100, Merck Millipore, AB5603). Slides were washed in PBS and incubated for 1 h at room temperature with Hoechst (2.5 µg mL^−1^; Sigma‐Aldrich, 33258) and the appropriate fluorophore‐conjugated secondary antibodies: goat anti‐mouse IgG1γ‐Alexa568 (1:100, Thermo Fisher Scientific, A‐21124), goat anti‐rabbit IgG‐Cy3 (1:800; Jackson ImmunoResearch, 111‐165‐144), goat anti‐rabbit IgG‐Alexa647 (1:100, Thermo Fisher Scientific, A‐21244), donkey anti‐guinea pig IgG‐Alexa488 (1:800; Jackson ImmunoResearch, 706‐545‐148), donkey anti‐rat‐Cy2 (1:100, Jackson ImmunoResearch, 712‐225‐150); and goat anti‐rat‐Cy3 (1:300, Jackson ImmunoResearch, 112‐165‐167). The slides were mounted with Fluoroshield (Sigma–Aldrich, F6182) and images were acquired with a Stellaris SP8 confocal microscope.

L1 DRG harvested from Becn1 cKO and crtl mice were fixed in 4% PFA for 2 h, washed in a solution of 0.01 M PBS (pH 7.2), and processed for paraffin embedding. In order to obtain a higher number of cellular bodies to analyze, DRG of each experimental group were pooled before embedding. Specimens were cut 10 µm thick using a Leica RM2125 microtome. After cutting, sections were dewaxed, permeabilized, and blocked for 1 h at room temperature with a solution of PBS 0.1% Triton, 1% Normal Donkey serum to saturate nonspecific signals. Sections were then incubated overnight at room temperature with the following primary antibodies: rat anti‐CD68 (1:100, Thermo Fisher Scientific, 14‐0681‐82) and rabbit anti‐Iba1 (1:100, Wako, 019–19741). The following day, slides were washed three times in PBS and incubated for 1 h at room temperature with the appropriate secondary antibodies: donkey anti‐rat IgG‐Cy3 (1:400, Jackson ImmunoResearch 712‐165‐153) and donkey anti‐rabbit IgG‐Alexa 488 (1:200, Jackson ImmunoResearch 711‐545‐152). Nuclei were stained with 4,6‐diamidino‐2‐phenylindole (DAPI 1:1000, Sigma 10236276001) in PBS. Finally, slides were mounted with a fluoromount medium (Sigma–Aldrich, F4680). Images were acquired using a Zeiss LSM800 confocal laser microscopy system (Zeiss, Jena, Germany).

### TUNEL Assay

The sciatic nerves were fixed in a 4% PFA solution in PBS for 1 h, then let equilibrate overnight at 4° C in a 30% sucrose solution in PBS. Sciatic nerves were then included in OCT and frozen in liquid nitrogen vapors. 10‐µm‐thick sciatic nerve cryosections were obtained and then processed by adapting the TUNEL‐assay manufacturer instructions (In situ cell death detection kit, TMR red, Roche, 12‐156‐792‐910). Briefly, slices were post‐fixed in 4% PFA in PBS for 15 min at room temperature, then washed twice in PBS for 5 min each. The tissue was then permeabilized with 0.1% Triton X‐100, 0.1% sodium citrate solution in PBS for 2 min at room temperature. Glass slides were rinsed twice in PBS, then incubated with the TUNEL reaction mixture with the addition of the nuclear stain Hoechst for 45 min at 37 °C in a dark humidified chamber. Slides were then rinsed three times in PBS and mounted with Fluoroshield to be imaged immediately at an epifluorescence (DMI4000, Leica) or confocal (Stellaris SP8) microscope.

### Western Blotting

Frozen sciatic or median nerve segments were mechanically disaggregated using a mortar and a pestle in liquid nitrogen vapors and then lysed with RIPA lysis buffer (50 mm Tris HCl, 150 mm NaCl, 1% IgePal, 0.5% Na deoxycholate, 0.1% SDS) supplemented with protease inhibitors (Sigma‐Aldrich, 04693132001) and phosphatase inhibitors (Sigma–Aldrich, P5726 and P0044). After 10 min centrifugation at maximum speed, the supernatant containing the nerve protein lysate was quantified with BCA Protein Kit Assay (Thermo‐Fisher, 23225). The same amount of proteins for each sample (10 µg) were separated by SDS‐PAGE using 4–12% or 12% polyacrylamide gels (Invitrogen, NP0342BOX). Samples were then blotted onto PVDF membrane (Thermo Fisher Scientific, 88518), blocked with 5% milk in 0.1% Tween 20 (Sigma‐Aldrich, P7949) in TBS (TBS‐T) and probed with primary antibodies in 2.5% milk in TBS‐T overnight at 4 °C. The following antibodies were used: rabbit anti‐Becn1 (1:1000, Cell Signaling Technology, 3738); rabbit anti‐MAG (1:1000, Cell Signaling Technology, 9043S); rabbit anti‐CNP (1:1000, Cell Signaling Technology, 5664S); rat anti‐MBP (1:2000, Abcam, ab7349); rabbit anti‐MPZ (1:5000, Abcam, ab31851); guinea pig anti‐p62 (1:800, Progen, GP62‐C); rat anti‐LAMP1 (1:1000, DSHB, 1D4B); rabbit ant LC3B (1:1000, Thermo Fisher Scientific, PA1‐16930); rat anti‐CD44 (1:1000, Invitrogen, 14‐0441‐82); rat anti‐S100A9 (1:1000, Abcam, AB105472); rat anti‐CD68 (1:800, AbD Serotec, MCA1957); rabbit anti‐phospho‐paxillin (1:1000, Chemicon, AB3837); rabbit anti‐phospho‐FAK (1:500, Santa Cruz Biotechnology, sc‐21831‐R); rabbit anti‐cdc42 (1:1000, GeneTex, 134588); rabbit anti‐phopho‐PIK3 p85/p55 (1:1000, Cell Signaling Technology, 4228S); rabbit anti‐myotubularin 1 (1:800, Novus, H00004534‐D01P); rabbit anti‐phopho‐PDK1 (1:1000, Cell Signaling Technology, 3438P); rabbit anti‐phospho‐AKT (T308) (1:1000, Cell Signaling Technology, 4056S); rabbit anti‐phospho‐mTOR (1:1000, Cell Signaling Technology, 5536S); mouse anti‐mTOR (1:1000, Cell Signaling Technology, 4517S); mouse anti‐vinculin (1:1500, Sigma‐Aldrich, V4505). After three washes in TBS‐T, membranes were incubated for 1 h at room temperature with the appropriate HRP‐conjugated secondary antibodies (Amersham Bioscience). Membranes were incubated with WesternBright Quantum HRP substrate kit reagents and the luminescent signal was detected by Amersham ImageQuant 800 biomolecular imager. Densitometric analysis was performed using the Fiji software.

### NMJ Analysis

Longitudinal 30‐µm‐thick tibialis anterior cross‐sections were fixed in 4% PFA for 15 min at room temperature, washed, and then permeabilized and saturated for 4 h at room temperature in a solution of 10% GS, 2% Triton‐X‐100 in PBS. Slices were incubated for 48 h at 4 °C with the following primary antibodies diluted in a solution of PBS supplemented with 1% GS and 2% Triton‐X‐100: α‐synaptophysin (1:50, Santa Cruz Biotechnology, sc‐9116) and α‐peripherin (1:200, Novus Biologicals, NB300‐137). Samples were then washed 3 times in PBS and incubated overnight at 4 °C with anti‐rabbit Cy2 (1:200, Jackson Immunoresearch) secondary antibody and Alexa Fluor 555 conjugate α‐bungarotoxin (α‐BTX) (1:1000, Invitrogen), diluted in PBS containing 1% GS and 0.02% Triton‐X‐100. After three washes in PBS, samples were mounted with Fluoroshield before imaging by a Leica Stellaris 5 confocal microscope coupled with the Leica Application Suite X (LASX) software. Z‐stacks with a 1‐µm interval of NMJs were taken with a 63x oil immersion objective, a 2.5x zoom, and a 512 × 512 frame size. For each sample, at least 35 NMJs were randomly acquired. The number of acetylcholine receptor (AChR) fragments for each NMJ was determined only for completely or almost completely planar NMJs by visual inspection of 3D projections of z‐stacks using the Fiji software. The innervation status was manually assessed by two different operators working in a blind fashion by visual inspection of z‐stacks using the LASX software. Only NMJs with a clear innervation status were included in the analysis. Based on their innervation status, NMJs were assigned to three different classes: fully innervated (pre‐ and post‐synaptic staining closely juxtaposed through the entire NMJ), partially innervated (post‐synaptic staining with some regions devoid of pre‐synaptic staining), and denervated (no pre‐synaptic staining overlying the post‐synaptic staining).

### RNA Extraction and Quantitative RT‐PCR

RNA extraction was performed from sciatic nerves, spinal cord, and dorsal root ganglia using TRIzol reagent (Invitrogen, 15596018), according to the manufacturer's instructions. Total RNA was quantified using a NanoDrop spectrophotometer and the same RNA amount per tissue were retrotranscribed with “High‐Capacity cDNA Reverse Transcription Kit” (Applied Biosystems), following manufacturer's instructions. Quantitative PCR was performed on a QuantStudio5 real‐time instrument (Applied Biosystems), using the SYBR‐green containing HOT FIREPol Blend Master Mix (Solis Biodyne). *Gapdh* and *TBP* were used as housekeeping genes for the analysis on spinal cord and DRG samples, respectively. Used primers are listed in Table S2 (Supporting Information).

### Transcriptomic Analysis

For microarray experiments, in vitro transcription, hybridization, and biotin labeling of RNA were performed according to the Mouse WT GeneChip Clariom S assay (Affymetrix, SantaClara, CA). CEL files were normalized using the robust multiarray averaging expression measure with Transcriptome Analysis Console (TAC Software v. 4.0.2.15, ThermoFisher Scientific, Waltham, MA, USA). Principal Component Analysis (PCA) was performed with the most 5000 variables genes selected on variance by R package (www-r-projects.org). Differentially expressed genes between control and Becn1 cKO mice were identified using the Significance Analysis of Microarray (SAM) algorithm coded in the samr R package^[^ ^101^ ^]^ for both P10 and 2‐month‐old time points. Estimated percentage of false‐positive predictions (i.e., False Discovery Rate, FDR) was obtained with 1000 permutations, and genes with an FDR < 0.05 were considered significant for both time points. Volcano plots were generated with the top 5000 variables genes used for SAM analysis. Deregulated genes (DEGs) at P10 and 2‐month‐old were used to perform Gene Set Enrichment Analysis (Gene Ontology Biological Process (GO‐BP), Reactome and Human Phenotype Ontology (HPO)) using ClusterProfiler package (v4.4.4)^[^ ^102^ ^]^ and compareCluster() function for biological theme comparison. Enriched terms were selected with p.adjusted <0.05 and BH correction. To find genes with a significant temporal expression changes between the two time points and significant differences between the experimental groups maSigPro (v. 1.60.0) package in R was used.^[^ ^103^ ^]^ The regression fit for each gene was calculated with a FDR<0.05 and Benjamini‐Hochbelg (BH) correction. R‐squared cut‐off level of 0.8 was used for the regression model. MClust() function was applied to compute the optimal clusters of genes with correlated expression patterns. A pheatmap package was used to obtained a heatmap of the 550 genes selected in time series analysis. Euclidean distane and Ward.d method were applied for clustering. Over‐representation analysis by using ClusterProfiler package (v4.4.4) were performed in specific gene clusters (GO‐BP and Reactome gene sets). Enriched terms were selected with p.adjusted <0.1 and BH correction. To reduce the complexity of terms in GO‐BP treeplot() function was used. Pairwise similarities of the enriched terms were calculated by the pairwise_termsim() function with default Jaccard's similarity index. The complete method was used for agglomeration in treeplot() function and to perform hierarchical clustering analysis. Expression data were deposited into the Gene Expression Omnibus (GEO) database under Series Accession Number GSE247435 and are accessible without restrictions.

### Images Analyses

The count of myelinated axons per area was performed manually on transversal sciatic nerve TEM images at low magnification, exploiting Fiji software. The analysis of Remak bundles and their axons was performed on transversal sciatic nerve TEM images at high magnification, by manually measuring bundle size, axons diameter and counting axons number through the Fiji software. For autophagic markers immunofluorescence analysis, randomly selected fields were acquired from each longitudinal sciatic nerve sample with 63x objective as a 6 planes stack (1 µm Z‐step). The integrated density was measured on maximum projections exploiting Fiji software. For CD68 count, longitudinal sciatic nerve cryosections were stained and randomly selected non‐overlapping fields were acquired with 40x objective as 10 planes stack (1 µm Z‐step). The number of CD68‐positive cells was manually counted on maximum intensity projection with the cell counter plugin of Fiji software. For the analysis of apoptosis, longitudinal sciatic nerve cryosections were processed with the TMR red kit, then randomly selected non‐overlapping field were acquired with a 40x objective. The number of TMR red‐positive nuclei was manually counted with the Fiji software. For proliferation analysis, randomly selected fields were acquired from each longitudinal sciatic nerve sample with 40x objective as a 4 planes stack (1 µm Z‐step). The number of positively stained nuclei and of the total nuclei (Hoechst‐stained) was manually counted from maximum projection images exploiting Fiji software. For MBP staining and the count of peripherin‐positive axons, the entire transversal sciatic nerve cryosection was acquired with the *navigator* function of the confocal microscope with a 40x objective. Motor axons were counted manually, excluding non‐countable regions and sensory axons present in Remak fibers bundles, then the number of axons was normalized only over the analyzed area (µm^2^).

### Statistical Analysis

All statistical data were analyzed using GraphPad Prism 10.1.1. Unpaired two‐tailed Student's *t*‐test or unpaired two‐tailed Mann‐Whitney test were used for comparisons between the two groups. One‐way and two‐way analysis of variants (ANOVA) were used for comparisons between more than two groups. Statistical significance was set at *P* < 0.05. All histograms displayed individual values and data were shown as mean ± SEM. The number of biological replicates, *n*, was always greater than three. The specific statistical test, sample size, and probability value for each experiment can be found in the related figure legend.

## Conflict of Interest

The authors declare no conflict of interest.

## Author Contributions

M.C. designed the study, acquired funding, and supervised the analysis. L.G. supervised mouse line production and performed most of the experiments and relative analyses. L.R. performed behavioral and immunofluorescence analyses during the revision. S.B., L.P., and A.C. performed and analyzed transcriptomic data; R.D. contributed to behavioral and biochemical analysis. G.R., F.Z., and L.M. designed, performed, and analyzed DRG studies. S.N. and A.M. performed electrophysiological analyses. S.C. contributed to immunofluorescence analysis. D.B. and P.B. managed mouse colonies; L.G. and M.C. wrote the original draft; all the other authors contributed to the final revision of the manuscript.

## Supporting information

Supporting Information

Supplemental Video 1

Supplemental Video 2

Supplemental Video 3

## Data Availability

The data that support the findings of this study are openly available in Gene Expression Omnibus at https://www.ncbi.nlm.nih.gov/geo, reference number 247435.

## References

[1] M. R. Freeman , Curr. Opin. Neurobiol. 2006, 16, 119. 16387489 10.1016/j.conb.2005.12.004

[2] K.‐A. Nave , B. D. Trapp , Annu. Rev. Neurosci. 2008, 31, 535. 18558866 10.1146/annurev.neuro.30.051606.094309

[3] C. Taveggia , G. Zanazzi , A. Petrylak , H. Yano , J. Rosenbluth , S. Einheber , X. Xu , R. M. Esper , J. A. Loeb , P. Shrager , M. V. Chao , D. L. Falls , L. Role , J. L. Salzer , Neuron 2005, 47, 681. 16129398 10.1016/j.neuron.2005.08.017 PMC2387056

[4] A. Woodhoo , M. B. D. Alonso , A. Droggiti , M. Turmaine , M. D'Antonio , D. B. Parkinson , D. K. Wilton , R. Al‐Shawi , P. Simons , J. Shen , F. Guillemot , F. Radtke , D. Meijer , M. L. Feltri , L. Wrabetz , R. Mirsky , K. R. Jessen , Nat. Neurosci. 2009, 12, 839. 19525946 10.1038/nn.2323 PMC2782951

[5] M. L. Feltri , Y. Poitelon , S. C. Previtali , Neuroscientist 2016, 22, 252. 25686621 10.1177/1073858415572361 PMC5181106

[6] K. R. Jessen , R. Mirsky , Nat. Rev. Neurosci. 2005, 6, 671. 16136171 10.1038/nrn1746

[7] C. B. Reed , M. L. Feltri , E. R. Wilson , J. Anat. 2022, 241, 1219. 34131911 10.1111/joa.13484 PMC8671569

[8] K.‐A. Nave , Nature 2010, 468, 244. 21068833 10.1038/nature09614

[9] S. Y. Jang , Y. K. Shin , S. Y. Park , J. Y. Park , S.‐H. Rha , J. K. Kim , H. J. Lee , H. T. Park , PLoS One 2015, 10, e0116624. 25581066 10.1371/journal.pone.0116624 PMC4291222

[10] J. Belgrad , R. De Pace , R. D. Fields , J. Neurosci. 2020, 40, 256. 31744863 10.1523/JNEUROSCI.1066-19.2019 PMC6948934

[11] M. Haidar , V. Timmerman , Front. Mol. Neurosci. 2017, 10, 143. 28553203 10.3389/fnmol.2017.00143 PMC5425483

[12] A. M. Rossor , J. M. Polke , H. Houlden , M. M. Reilly , Nat. Rev. Neurol. 2013, 9, 562. 24018473 10.1038/nrneurol.2013.179

[13] J. R. Edgar , A. K. Ho , M. Laurá , R. Horvath , M. M. Reilly , J. P. Luzio , R. C. Roberts , Acta Neuropathol. Commun. 2020, 8, 165. 33059769 10.1186/s40478-020-01043-z PMC7559459

[14] R. Markworth , M. Bähr , K. Burk , Front. Mol. Neurosci. 2021, 14, 695294. 34483837 10.3389/fnmol.2021.695294 PMC8415527

[15] A. Ruck , J. Attonito , K. T. Garces , L. Núnez , N. J. Palmisano , Z. Rubel , Z. Bai , K. C. Q. Nguyen , L. Sun , B. D. Grant , D. H. Hall , A. Meléndez , Autophagy 2011, 7, 386. 21183797 10.4161/auto.7.4.14391 PMC3108013

[16] E. Wirawan , S. Lippens , T. Vanden Berghe , A. Romagnoli , G. M. Fimia , M. Piacentini , P. Vandenabeele , Autophagy 2012, 8, 6. 22170155 10.4161/auto.8.1.16645

[17] N. C. McKnight , Y. Zhong , M. S. Wold , S. Gong , G. R. Phillips , Z. Dou , Y. Zhao , N. Heintz , W.‐X. Zong , Z. Yue , PLoS Genet. 2014, 10, e1004626. 25275521 10.1371/journal.pgen.1004626 PMC4183436

[18] S. Noguchi , S. Honda , T. Saitoh , H. Matsumura , E. Nishimura , S. Akira , S. Shimizu , Commun. Biol. 2019, 2, 37. 30701202 10.1038/s42003-018-0279-0 PMC6347619

[19] H. Tang , S. Sebti , R. Titone , Y. Zhou , C. Isidoro , T. S. Ross , H. Hibshoosh , G. Xiao , M. Packer , Y. Xie , B. Levine , EBioMedicine 2015, 2, 255. 25825707 10.1016/j.ebiom.2015.01.008 PMC4376376

[20] G. Valente , F. Morani , G. Nicotra , N. Fusco , C. Peracchio , R. Titone , O. Alabiso , R. Arisio , D. Katsaros , C. Benedetto , C. Isidoro , Biomed Res. Int. 2014, 2014, 1. 10.1155/2014/462658 PMC4127242 25136588

[21] H. Zhu , L. He , Curr. Cardiol. Rev. 2015, 11, 229. 25373623 10.2174/1573403X10666141106104606 PMC4558354

[22] P. A. Jaeger , T. Wyss‐Coray , Arch. Neurol. 2010, 67, 1181. 20937944 10.1001/archneurol.2010.258

[23] G. Bieri , K. M. Lucin , C. E. O'Brien , H. Zhang , S. A. Villeda , T. Wyss‐Coray , Mol. Neurodegenerat. 2018, 13, 68. 10.1186/s13024-018-0302-4 PMC6310967 30594228

[24] Z. Yue , S. Jin , C. Yang , A. J. Levine , N. Heintz , Proc. Nat. Acad. Sci. USA 2003, 100, 15077. 14657337 10.1073/pnas.2436255100 PMC299911

[25] M. L. Feltri , D. G. Porta , S. C. Previtali , A. Nodari , B. Migliavacca , A. Cassetti , A. Littlewood‐Evans , L. F. Reichardt , A. Messing , A. Quattrini , U. Mueller , L. Wrabetz , J. Cell Biol. 2002, 156, 199. 11777940 10.1083/jcb.200109021 PMC2173589

[26] N. C. McKnight , Z. Yue , Curr. Pathobiol. Rep. 2013, 1, 231. 24729948 10.1007/s40139-013-0028-5 PMC3979578

[27] K. R. Jessen , R. Mirsky , Front. Mol. Neurosci. 2019, 12, 69. 30971890 10.3389/fnmol.2019.00069 PMC6443887

[28] A. Balakrishnan , L. Belfiore , T.‐H. Chu , T. Fleming , R. Midha , J. Biernaskie , C. Schuurmans , Front. Mol. Neurosci. 2021, 13, 608442. 33568974 10.3389/fnmol.2020.608442 PMC7868393

[29] J. L. Salzer , Cold Spring Harb Perspect. Biol. 2015, 7, a020529. 26054742 10.1101/cshperspect.a020529 PMC4526746

[30] S. L. Roberts , X.‐P. Dun , R. D. S. Doddrell , T. Mindos , L. K. Drake , M. W. Onaitis , F. Florio , A. Quattrini , A. C. Lloyd , M. D'Antonio , D. B. Parkinson , Development 2017, 144, 3114. 28743796 10.1242/dev.150656 PMC5611958

[31] F. Florio , C. Ferri , C. Scapin , M. L. Feltri , L. Wrabetz , M. D'Antonio , J. Neurosci. 2018, 38, 4275. 29610440 10.1523/JNEUROSCI.0201-18.2018 PMC6596005

[32] K. R. Jessen , R. Mirsky , Glia 2008, 56, 1552. 18803323 10.1002/glia.20761

[33] P. J. Arthur‐Farraj , M. Latouche , D. K. Wilton , S. Quintes , E. Chabrol , A. Banerjee , A. Woodhoo , B. Jenkins , M. Rahman , M. Turmaine , G. K. Wicher , R. Mitter , L. Greensmith , A. Behrens , G. Raivich , R. Mirsky , K. R. Jessen , Neuron 2012, 75, 633. 22920255 10.1016/j.neuron.2012.06.021 PMC3657176

[34] H. T. Park , J. K. Kim , N. Tricaud , Glia 2019, 67, 571. 30378179 10.1002/glia.23509

[35] J. L. Shadrach , W. M. Stansberry , A. M. Milen , R. E. Ives , E. A. Fogarty , A. Antonellis , B. A. Pierchala , iScience 2021, 24, 102700. 34235408 10.1016/j.isci.2021.102700 PMC8246596

[36] Y. Zhang , L. Xu , X. Li , Z. Chen , J. Chen , T. Zhang , X. Gu , J. Yang , iScience 2022, 25, 104917. 36051182 10.1016/j.isci.2022.104917 PMC9424597

[37] A. Kritis , D. Kapoukranidou , B. Michailidou , A. Hatzisotiriou , M. Albani , Hippokratia 2010, 14, 37. 20411058 PMC2843569

[38] G. Ronchi , G. Gambarotta , F. Di Scipio , P. Salamone , A. E. Sprio , F. Cavallo , I. Perroteau , G. N. Berta , S. Geuna , PLoS One 2013, 8, e56282. 23437108 10.1371/journal.pone.0056282 PMC3578860

[39] M. Y. Jin , T. E. Weaver , A. Farris , M. Gupta , A. Abd‐Elsayed , Biomedicines 2023, 11, 1145. 37189763 10.3390/biomedicines11041145 PMC10135453

[40] K. Ren , R. Dubner , Nat. Med. 2010, 16, 1267. 20948535 10.1038/nm.2234 PMC3077564

[41] M. Bosch‐Queralt , R. Fledrich , R. M. Stassart , Neurobiol. Disease 2023, 176, 105952. 10.1016/j.nbd.2022.105952 36493976

[42] K. Meller , Cell Tissue Res. 1989, 256, 283. 2731217 10.1007/BF00218885

[43] I. P. Johnson , T. A. Sears , Neuroscience 2013, 228, 163. 23079627 10.1016/j.neuroscience.2012.10.015

[44] M. J. Groves , F. Scaravilli , in Peripheral Neuropathy, Elsevier, Amsterdam New York, 2005, p. 683.

[45] S. L. Martin , A. J. Reid , A. Verkhratsky , V. Magnaghi , A. Faroni , Neural. Regen. Res. 2019, 14, 939. 30761997 10.4103/1673-5374.250566 PMC6404509

[46] W. Renthal , I. Tochitsky , L. Yang , Y.‐C. Cheng , E. Li , R. Kawaguchi , D. H. Geschwind , C. J. Woolf , Neuron 2020, 108, 128. 32810432 10.1016/j.neuron.2020.07.026 PMC7590250

[47] A. L. Marat , V. Haucke , EMBO J. 2016, 35, 561. 26888746 10.15252/embj.201593564 PMC4801949

[48] Y. Posor , W. Jang , V. Haucke , Nat. Rev. Mol. Cell Biol. 2022, 23, 797. 35589852 10.1038/s41580-022-00490-x PMC9117997

[49] F. Blondeau , J. Laporte , S. Bodin , G. Superti‐Furga , B. Payrastre , J. L. Mandel , Hum. Mol. Genet. 2000, 9, 2223. 11001925 10.1093/oxfordjournals.hmg.a018913

[50] A. Levina , K. D. Fleming , J. E. Burke , T. A. Leonard , Nat. Commun. 2022, 13, 1874. 35387990 10.1038/s41467-022-29368-4 PMC8986801

[51] M. A. Chernousov , W.‐M. Yu , Z.‐L. Chen , D. J. Carey , S. Strickland , Glia 2008, 56, 1498. 18803319 10.1002/glia.20740

[52] M. L. Feltri , U. Suter , J. B. Relvas , Glia 2008, 56, 1508. 18803320 10.1002/glia.20752 PMC2615182

[53] P. Berger , A. Niemann , U. Suter , Glia 2006, 54, 243. 16856148 10.1002/glia.20386

[54] J. A. Gomez‐Sanchez , L. Carty , M. Iruarrizaga‐Lejarreta , M. Palomo‐Irigoyen , M. Varela‐Rey , M. Griffith , J. Hantke , N. Macias‐Camara , M. Azkargorta , I. Aurrekoetxea , V. G. De Juan , H. B. J. Jefferies , P. Aspichueta , F. Elortza , A. M. Aransay , M. L. Martínez‐Chantar , F. Baas , J. M. Mato , R. Mirsky , A. Woodhoo , K. R. Jessen , J. Cell Biol. 2015, 210, 153. 26150392 10.1083/jcb.201503019 PMC4494002

[55] R. A. Frake , T. Ricketts , F. M. Menzies , D. C. Rubinsztein , J. Clin. Invest. 2015, 125, 65. 25654552 10.1172/JCI73944 PMC4382230

[56] F. M. Menzies , A. Fleming , D. C. Rubinsztein , Nat. Rev. Neurosci. 2015, 16, 345. 25991442 10.1038/nrn3961

[57] Y. Son , Y. K. Cho , A. Saha , H.‐J. Kwon , J.‐H. Park , M. Kim , Y.‐S. Jung , S.‐N. Kim , C. Choi , J.‐K. Seong , R. B. Burl , J. G. Granneman , Y.‐H. Lee , Mol. Metab. 2020, 39, 101005. 32344065 10.1016/j.molmet.2020.101005 PMC7235646

[58] M. Simons , J. Trotter , Curr. Opin. Neurobiol. 2007, 17, 533. 17923405 10.1016/j.conb.2007.08.003

[59] G. Chen , Z. Zhang , Z. Wei , Q. Cheng , X. Li , W. Li , S. Duan , X. Gu , Glia 2012, 60, 295. 22042600 10.1002/glia.21263

[60] A. M. Logan , A. E. Mammel , D. C. Robinson , A. L. Chin , A. F. Condon , F. L. Robinson , Glia 2017, 65, 1452. 28617998 10.1002/glia.23173 PMC5818149

[61] D. Gerber , M. Ghidinelli , E. Tinelli , C. Somandin , J. Gerber , J. A. Pereira , A. Ommer , G. Figlia , M. Miehe , L. G. Nägeli , V. Suter , V. Tadini , P. N. Sidiropoulos , C. Wessig , K. V. Toyka , U. Suter , eLife 2019, 8, e42404. 30648534 10.7554/eLife.42404 PMC6335055

[62] A.‐C. Durieux , B. Prudhon , P. Guicheney , M. Bitoun , J. Mol. Med. 2010, 88, 339. 20127478 10.1007/s00109-009-0587-4

[63] A. Raimondi , S. M. Ferguson , X. Lou , M. Armbruster , S. Paradise , S. Giovedi , M. Messa , N. Kono , J. Takasaki , V. Cappello , E. O'Toole , T. A. Ryan , P. De Camilli , Neuron 2011, 70, 1100. 21689597 10.1016/j.neuron.2011.04.031 PMC3190241

[64] S. M. Ferguson , P. De Camilli , Nat. Rev. Mol. Cell Biol. 2012, 13, 75. 22233676 10.1038/nrm3266 PMC3519936

[65] D. Klein , R. Martini , Brain Res. 2016, 1641, 130. 26631844 10.1016/j.brainres.2015.11.033

[66] N. Tricaud , H. T. Park , Cell. Mol. Life Sci. 2017, 74, 4049. 28600652 10.1007/s00018-017-2565-2 PMC5641270

[67] G. K. Tofaris , P. H. Patterson , K. R. Jessen , R. Mirsky , J. Neurosci. 2002, 22, 6696. 12151548 10.1523/JNEUROSCI.22-15-06696.2002 PMC6758146

[68] J. A. Lindborg , M. Mack , R. E. Zigmond , J. Neurosci. 2017, 37, 10258. 28912156 10.1523/JNEUROSCI.2085-17.2017 PMC5656991

[69] A. L. Kalinski , C. Yoon , L. D. Huffman , P. C. Duncker , R. Kohen , R. Passino , H. Hafner , C. Johnson , R. Kawaguchi , K. S. Carbajal , J. S. Jara , E. Hollis , D. H. Geschwind , B. M. Segal , R. J. Giger , eLife 2020, 9, e60223. 33263277 10.7554/eLife.60223 PMC7735761

[70] J. M. Vicencio , C. Ortiz , A. Criollo , A. W. E. Jones , O. Kepp , L. Galluzzi , N. Joza , I. Vitale , E. Morselli , M. Tailler , M. Castedo , M. C. Maiuri , J. Molgó , G. Szabadkai , S. Lavandero , G. Kroemer , Cell Death Differ. 2009, 16, 1006. 19325567 10.1038/cdd.2009.34

[71] L. S. Sherman , T. A. Rizvi , S. Karyala , N. Ratner , J. Cell Biol. 2000, 150, 1071. 10973996 10.1083/jcb.150.5.1071 PMC2175255

[72] H. Tsujino , E. Kondo , T. Fukuoka , Y. Dai , A. Tokunaga , K. Miki , K. Yonenobu , T. Ochi , K. Noguchi , Mol. Cell. Neurosci. 2000, 15, 170. 10673325 10.1006/mcne.1999.0814

[73] K. R. Moss , T. S. Bopp , A. E. Johnson , A. Höke , Neurosci. Lett. 2021, 744, 135595. 33359733 10.1016/j.neulet.2020.135595 PMC7852893

[74] H. Franssen , Adv. Exp. Med. Biol. 2019, 1190, 85. 31760640 10.1007/978-981-32-9636-7_7

[75] B. Beirowski , E. Babetto , J. P. Golden , Y.‐J. Chen , K. Yang , R. W. Gross , G. J. Patti , J. Milbrandt , Nat. Neurosci. 2014, 17, 1351. 25195104 10.1038/nn.3809 PMC4494117

[76] J. Groh , H. C. Friedman , N. Orel , C. W. Ip , S. Fischer , I. Spahn , E. Schäffner , M. Hörner , D. Stadler , M. Buttmann , C. Varallyay , L. Solymosi , M. Sendtner , A. C. Peterson , R. Martini , Hum. Mol. Genet. 2016, 25, 4686. 28173160 10.1093/hmg/ddw296

[77] L. Bosco , Y. M. Falzone , S. C. Previtali , Brain Sci. 2021, 11, 1237. 34573256 10.3390/brainsci11091237 PMC8465478

[78] V. Fridman , M. A. Saporta , Neurotherapeutics 2021, 18, 2236. 34750751 10.1007/s13311-021-01145-z PMC8804145

[79] E. J. Hutton , L. Carty , M. Laurá , H. Houlden , M. P. T. Lunn , S. Brandner , R. Mirsky , K. Jessen , M. M. Reilly , J. Peripher. Nervous Syst. 2011, 16, 295. 10.1111/j.1529-8027.2011.00360.x 22176144

[80] R. Martini , D. Klein , J. Groh , Am. J. Pathol. 2013, 183, 655. 23831295 10.1016/j.ajpath.2013.06.002

[81] J. Hantke , L. Carty , L. J. Wagstaff , M. Turmaine , D. K. Wilton , S. Quintes , M. Koltzenburg , F. Baas , R. Mirsky , K. R. Jessen , Brain 2014, 137, 2922. 25216747 10.1093/brain/awu257 PMC4208468

[82] L. Daboussi , G. Costaguta , M. Gullo , N. Jasinski , V. Pessino , B. O'Leary , K. Lettieri , S. Driscoll , S. L. Pfaff , Cell Rep. 2023, 42, 113282. 38007688 10.1016/j.celrep.2023.113282 PMC11034927

[83] K. M. Brennan , Y. Bai , M. E. Shy , Neurosci. Lett. 2015, 596, 14. 25625223 10.1016/j.neulet.2015.01.059

[84] R. Martini , K. V. Toyka , Lancet Neurol. 2004, 3, 457. 15261606 10.1016/S1474-4422(04)00822-1

[85] R. Sivera Mascaró , T. García Sobrino , A. Horga Hernández , A. L. Pelayo Negro , A. Alonso Jiménez , A. Antelo Pose , M. D. Calabria Gallego , C. Casasnovas , C. A. Cemillán Fernández , J. Esteban Pérez , M. Fenollar Cortés , M. Frasquet Carrera , M. P. Gallano Petit , A. Giménez Muñoz , G. Gutiérrez Gutiérrez , A. Gutiérrez Martínez , R. Juntas Morales , N. L. Ciano‐Petersen , P. L. Martínez Ulloa , S. Mederer Hengstl , E. Millet Sancho , F. J. Navacerrada Barrero , F. E. Navarrete Faubel , J. Pardo Fernández , S. I. Pascual Pascual , J. Pérez Lucas , J. Pino Mínguez , M. Rabasa Pérez , M. Sánchez González , J. Sotoca , et al., Neurologia 2024, S2173. 10.1016/j.nrleng.2024.02.008 38431252

[86] M. B. Bunge , M. B. Clark , A. C. Dean , C. F. Eldridge , R. P. Bunge , Ann. N. Y. Acad. Sci. 1990, 580, 281. 2337301 10.1111/j.1749-6632.1990.tb17937.x

[87] P. Maurel , J. L. Salzer , J. Neurosci. 2000, 20, 4635. 10844033 10.1523/JNEUROSCI.20-12-04635.2000 PMC6772460

[88] B. A. Heller , M. Ghidinelli , J. Voelkl , S. Einheber , R. Smith , E. Grund , G. Morahan , D. Chandler , L. Kalaydjieva , F. Giancotti , R. H. King , A. N. Fejes‐Toth , G. Fejes‐Toth , M. L. Feltri , F. Lang , J. L. Salzer , J. Cell Biol. 2014, 204, 1219. 24687281 10.1083/jcb.201307057 PMC3971744

[89] M. Grove , P. J. Brophy , J. Neurosci. 2014, 34, 13422. 25274820 10.1523/JNEUROSCI.1764-14.2014 PMC4180476

[90] L. M. Chen , D. Bailey , C. Fernandez‐Valle , J. Neurosci. 2000, 20, 3776. 10804218 10.1523/JNEUROSCI.20-10-03776.2000 PMC6772705

[91] Y. Benninger , T. Thurnherr , J. A. Pereira , S. Krause , X. Wu , A. Chrostek‐Grashoff , D. Herzog , K.‐A. Nave , R. J. M. Franklin , D. Meijer , C. Brakebusch , U. Suter , J. B. Relvas , J. Cell Biol. 2007, 177, 1051. 17576798 10.1083/jcb.200610108 PMC2064365

[92] E. Domènech‐Estévez , H. Baloui , X. Meng , Y. Zhang , K. Deinhardt , J. L. Dupree , S. Einheber , R. Chrast , J. L. Salzer , J. Neurosci. 2016, 36, 4506. 27098694 10.1523/JNEUROSCI.3521-15.2016 PMC4837684

[93] L. F. Castelnovo , V. Bonalume , S. Melfi , M. Ballabio , D. Colleoni , V. Magnaghi , Neural. Regen. Res. 2017, 12, 1013. 28852375 10.4103/1673-5374.211172 PMC5558472

[94] A. Alvarez‐Prats , I. Bjelobaba , Z. Aldworth , T. Baba , D. Abebe , Y. J. Kim , S. S. Stojilkovic , M. Stopfer , T. Balla , Cell Rep. 2018, 23, 2881. 29874576 10.1016/j.celrep.2018.05.019 PMC7268203

[95] T. Baba , A. Alvarez‐Prats , Y. J. Kim , D. Abebe , S. Wilson , Z. Aldworth , M. A. Stopfer , J. Heuser , T. Balla , Proc. Natl. Acad. Sci. USA 2020, 117, 28102. 33106410 10.1073/pnas.2007432117 PMC7668188

[96] I. Vaccari , G. Dina , H. Tronchère , E. Kaufman , G. Chicanne , F. Cerri , L. Wrabetz , B. Payrastre , A. Quattrini , L. S. Weisman , M. H. Meisler , A. Bolino , PLoS Genet. 2011, 7, e1002319. 22028665 10.1371/journal.pgen.1002319 PMC3197679

[97] I. Vaccari , A. Carbone , S. C. Previtali , Y. A. Mironova , V. Alberizzi , R. Noseda , C. Rivellini , F. Bianchi , U. Del Carro , M. D'Antonio , G. M. Lenk , L. Wrabetz , R. J. Giger , M. H. Meisler , A. Bolino , Hum. Mol. Genet. 2015, 24, 383. 25187576 10.1093/hmg/ddu451 PMC4275070

[98] C. Bucci , O. Bakke , C. Progida , Prog. Neurobiol. 2012, 99, 191. 22465036 10.1016/j.pneurobio.2012.03.003 PMC3514635

[99] M. Juneja , J. Burns , M. A. Saporta , V. Timmerman , J. Neurol. Neurosurg. Psychiatry 2019, 90, 58. 30018047 10.1136/jnnp-2018-318834 PMC6327864

[100] S. J. Tunster , Biol. Sex Diff. 2017, 8, 31. 10.1186/s13293-017-0154-6 PMC5645908 29041956

[101] V. G. Tusher , R. Tibshirani , G. Chu , Proc. Nat. Acad. Sci. USA 2001, 98, 5116. 11309499 10.1073/pnas.091062498 PMC33173

[102] G. Yu , L.‐G. Wang , Y. Han , Q.‐Y. He , OMICS: A J. Integrat. Biol. 2012, 16, 284. 10.1089/omi.2011.0118 PMC3339379 22455463

[103] A. Conesa , M. J. Nueda , A. Ferrer , M. Talón , Bioinformatics 2006, 22, 1096. 16481333 10.1093/bioinformatics/btl056

