# Supplementary material for: Schwann Cell‐Specific Ablation of Beclin 1 Impairs Myelination and Leads to Motor and Sensory Neuropathy in Mice
Source: Adv Sci (Weinh). 2024 Dec 16;12(5):2308965. doi: 10.1002/advs.202308965 (PMC11792035; doi:10.1002/advs.202308965)
Supplement: Supplementary file 1 — Supporting Information [file ADVS-12-2308965-s001.docx]

Supporting Information

**Schwann Cell-Specific Ablation of Beclin 1 Impairs Myelination and Leads to Motor and Sensory Neuropathy in Mice**

*Lisa Gambarotto, Loris Russo, Silvia Bresolin, Luca Persano, Rachele D’Amore, Giulia Ronchi, Federica Zen, Luisa Muratori, Alice Cani, Samuele Negro, Aram Megighian, Sonia Calabrò, Paola Braghetta, Dario Bizzotto and Matilde Cescon**

**Supplementary Material 1.** Three representative videos showing **(a)** a control 2-month-old mouse, **(b)** a *Becn1* cKO 2-month-old mouse with involuntary tremors and **(c)** a 6-month-old *Becn1* cKO mouse with involuntary tremors, difficulties in movements and hindlimb paresis.

**Supplementary Figure S1. (a)** Schematic representation of *Becn1* gene, either floxed (first line) or upon Cre-recombinase excision, with the consequent ablation of exons 4 to 7 (second line). Exons are represented as black boxes. **(b)** Normalized mRNA levels from RT-qPCR analyses on two different regions (exons 2-3 and exons 5-6) of *Becn1* transcript in cerebellum mRNA extract of 6-month-old control and *Becn1* cKO mice (*n* = 3 mice each group; ns, not significant; Mann-Whitney U test). **(c)** Representative western blot images of Beclin 1 (Becn1) and myelin proteins on total sciatic nerves protein extracts compared to cerebellum protein extracts in 6-month-old control, *Becn1* cHet and *Becn1* cKO mice. Vinculin (VCL) was used as loading control. ctrl, control.

**Supplementary Figure S2. (a)** Representative images of toluidine blue-stained semithin sciatic nerve transversal sections from P10, 2-month-old and 6-month-old *Becn1* cHet mice. Scale bar = 40 µm. **(b)** Representative transmission electron microscopy images of sciatic nerve transversal sections from P10, 2-month-old and 6-month-old *Becn1* cHet mice. Scale bar = 10 µm. **(c)** Representative western blot images and relative densitometric quantifications of myelin proteins on total median nerves protein extracts of 6-month-old control and *Becn1* cKO mice. Vinculin (VCL) was used as loading control (*n* = 5-6 mice each group; *, *P*<0.05; Kruskal-Wallis test with Dunn’s test for multiple comparisons). **(d)** Representative confocal immunofluorescence images for myelin basic protein (MBP, green) and peripherin (magenta) on entire transversal cryosection of sciatic nerves from 2-month-old control and *Becn1* cKO mice. Nuclei were counterstained with Hoechst (H, blue). Scale bar = 100 µm. Enlarged view of the highlighted area is provided on the right, with scale bar = 50 µm. **(e)**  Representative transmission electron microscopy images of optic nerve transversal sections from 6-month-old control, *Becn1* cHet and *Becn1* cKO mice. Scale bar = 2 µm. 2mo, 2-month-old; 6mo, 6-month-old; ctrl, control; A.U., arbitrary units.

**Supplementary Figure S3. (a)** Principal Component Analysis (PCA) of the most 5000 variables transcript clusters in P10 and 2-month-old control and *Becn1* cKO mice sciatic nerves. **(b,c)** Volcano plot of DEGs obtained from SAM analysis in *Becn1* cKO versus control mice in P10 (b) and 2-month-old samples (c). All significantly *Becn1* cKO deregulated genes are in red (upregulated) and blue (downregulated). **(d-e)** Venn-diagrams showing the numbers of down- (d) and up-regulated (e) transcripts in P10 and 2-month-old *Becn1* cKO mice sciatic nerves. **(f)** Trend gene expression of control and *Becn1* cKO genes through time for the median profile of the indicated gene numbers belonging to the nine generated time-series clusters. Red squares indicate the analyzed clusters (3 and 6). In x-axis time factor; in y-axis gene expression; line colors indicate experimental groups and average gene expression of each time-group. Dots represent individual samples. **(g)** Heatmap displaying the individual expression of the 550 genes belonging to selected clusters (red squared in (f)) in time-series analysis. 2mo, 2-month-old; ctrl, control.

**Supplementary Figure S4. (a)** Representative confocal immunofluorescence images for SOX10 (red) and SOX2 (green) on longitudinal cryosection of sciatic nerves from 2-month-old control and *Becn1* cKO mice. Nuclei were counterstained with Hoechst (blue); scale bar = 50 µm. **(b)** Representative transmission electron microscopy images of sciatic nerve transversal sections from P21 control and *Becn1* cKO mice. Scale bar = 10 µm. Inset for details of myelin membranes undergoing degradation are provided with scale bar = 2 µm. **(c)** Representative transmission electron microscopy images of sciatic nerve transversal sections from P21 *Becn1* cKO mice showing myelin exocytosis (i) and SCs and axons contacted by macrophages. Yellow asterisks indicate myelin membranes into vesicles; red arrowheads indicate macrophage podia within SC basal lamina. Scale bar = 1 µm. Ax, axon; ctrl, control; M, macrophage; SC, Schwann cell.

**Supplementary Figure S5. (a)** Representative confocal immunofluorescence images for Iba1 (green) and CD68 (yellow) on L1 DRG from 2-month-old control and *Becn1* cKO mice. Nuclei were counterstained with DAPI (blue). Arrows indicate colocalization for CD68 and Iba1 immune cell markers. Scale bar = 20 µm. ctrl, control.

**Supplementary Figure S6. (a)** Rotarod performance of 5-month-old control and *Becn1* cKO mice (n = 8 control; n = 4 *Becn1* cKO mice; **, *P*<0.05; unpaired two-tailed Student’s t-test). **(b)** Representative confocal images of NMJs labeled with BTX (red) and antibodies to synaptophysin (SYP) and peripherin (green) in tibialis anterior muscles of 2-month-old control and *Becn1* cKO mice. Three different representative innervation patterns are shown: fully innervated, partially innervated and denervated endplates. **(c)** Quantification of the proportion of innervated, partially innervated and denervated endplates in tibialis anterior muscles of 2-month-old control and *Becn1* cKO mice (multiple unpaired two-tailed Student’s T tests; n= 3 mice, each group). Scale bar = 10 µm. **(d)** Representative images of 555-bungarotoxin (BTX) stained NMJs in longitudinal cryosections of tibialis anterior muscles from 2-month-old control and *Becn1* cKO mice. White arrowheads highlight AChR cluster fragments. **(e)** Quantification of the average number of fragments per NMJ in control versus *Becn1* cKO 2-month-old mice (Mann-Whitney U test; *n* = 116-144 NMJs from 3 mice, each group). **(f)** Percentage of total NMJs displaying the number of fragments reported in the *x*-axis in control versus *Becn1* cKO 2-month-old mice (Mann-Whitney U test; *n* = 3 mice, each group). ctrl, control.

Supplementary Figure S7. (a) Representative confocal immunofluorescence images for PI3P (red) staining on longitudinal cryosections of sciatic nerves from P10 control and *Becn1* cKO mice. Nuclei were counterstained with Hoechst (blue); scale bar = 50 µm. ctrl, control.
